# PRM-MS Quantitative Analysis of Isomeric *N*-Glycopeptides Derived from Human Serum Haptoglobin of Patients with Cirrhosis and Hepatocellular Carcinoma

**DOI:** 10.3390/metabo11080563

**Published:** 2021-08-23

**Authors:** Cristian D. Gutierrez Reyes, Yifan Huang, Mojgan Atashi, Jie Zhang, Jianhui Zhu, Suyu Liu, Neehar D. Parikh, Amit G. Singal, Jianliang Dai, David M. Lubman, Yehia Mechref

**Affiliations:** 1 Department of Chemistry and Biochemistry, Texas Tech University, Lubbock, TX 79409, USA; Cristian.d.gutierrez-reyes@ttu.edu (C.D.G.R.); yifan.huang@ttu.edu (Y.H.); Mojgan.athasi@ttu.edu (M.A.); 2 Department of Surgery, The University of Michigan, Ann Arbor, MI 48109, USA; Jzhangte@umich.edu (J.Z.); Jianhuiz@umich.edu (J.Z.); Dmlubman@umich.edu (D.M.L.); 3 Department of Biostatistics, University of Texas MD Anderson Cancer Center, Houston, TX 77030, USA; Syliu@mdanderson.org (S.L.); Jdai4@mdanderson.org (J.D.); 4 Division of Gastroenterology and Hepatology, The University of Michigan, Ann Arbor, MI 48109, USA; ndparikh@med.umich.edu; 5 Division of Digestive and Liver Diseases, University of Texas Southwestern Medical Center, Dallas, TX 75390, USA; Amit.singal@utsouthwestern.edu

**Keywords:** PRM, *N*-glycopeptides, haptoglobin, cirrhosis, hepatocellular carcinoma (HCC)

## Abstract

Currently, surveillance strategies have inadequate performance for cirrhosis and early detection of hepatocellular carcinoma (HCC). The glycosylation of serum haptoglobin has shown to have significant differences between cirrhosis and HCC, thus can be used for diagnosis. We performed a comprehensive liquid chromatography—parallel reaction monitoring—mass spectrometry (LC-PRM-MS) approach, where a targeted parallel reaction monitoring (PRM) strategy was coupled to a powerful LC system, to study the site-specific isomerism of haptoglobin (Hp) extracted from cirrhosis and HCC patients. We found that our strategy was able to identify a large number of isomeric *N*-glycopeptides, mainly located in the Hp glycosylation site Asn207. Four *N*-glycopeptides were found to have significant changes in abundance between cirrhosis and HCC samples (*p* < 0.05). Strategic combinations of the significant *N*-glycopeptides, either with alpha-fetoprotein (AFP) or themselves, better estimate the areas under the curve (AUC) of their respective receiver operating characteristic (ROC) curves with respect to AFP. The combination of AFP with the isomeric sialylated fucosylated *N*-glycopeptides Asn207 + 5-6-1-2 and Asn207 + 5-6-1-3, resulted with an AUC value of 0.98, while the AUC value for AFP alone was 0.85. When comparing cirrhosis vs. early HCC, the isomeric *N*-glycopeptide Asn207 + 5-6-0-1 better estimated AUC with respect to AFP (AUC_AFP_ = 0.81, and AUC_Asn207_ + 5-6-0-1 = 0.88, respectively).

## 1. Introduction

Glycosylation alterations of serum proteins are commonly associated with the development of several cancer types; therefore, changes in protein glycosylation may play an important role in disease diagnosis [1,2,3]. The development of accurate markers for the early stages of cancer types with high mortality rates, such as hepatocellular carcinoma (HCC) [4], may provide opportunities for more effective patient treatments [5]. Most HCCs develop in the setting of cirrhosis [6]; however, current early detection strategies, using abdominal ultrasound and alpha-fetoprotein (AFP), have inadequate sensitivity and specificity for early detection [7]. The performance of ultrasound is further diminished in patients with nonalcoholic steatohepatitis (NASH), associated with central obesity, and the fastest growing cause of HCC in the US [8]. The male-to-female incidence ratio of HCC varies between populations, in a ratio of 2:1 and 4:1. The excess risk of HCC in men is explained by the high prevalence of factors such as hepatitis B virus infection (HBV), alcohol use, and smoking [9]. An accurate marker-based strategy, particularly in patients with NASH, has the potential to improve HCC-related outcomes.

In recent years, glycomic studies of serum haptoglobin have identified important isomeric structures, most of them being sialylated fucosylated glycans, with significant differences in abundance between patients with cirrhosis and HCC [1,3]. Analyses of haptoglobin *N*-glycopeptides have described the heterogeneity of their glycosylation sites, and have identified potential sialylated fucosylated *N*-glycopeptide structures as potential biomolecules to differentiate between both diseases [10,11,12]. According to these studies, glycoproteomic analysis, focused on an extensive determination of isomeric *N*-glycopeptide structures, could increase the possibility of finding haptoglobin site-specific *N*-glycopeptides that can differentiate between cirrhosis and the early stages of HCC. Moreover, the glycosylation site information, obtained by glycoproteomic analysis, would be an important factor in understanding how aberrant glycosylation is related to disease progression [13].

The structural identification and quantitation of the isomeric site-specific glycosylation of haptoglobin remains challenging, due to the low abundance of important sialylated fucosylated structures [1,2,10,14]. In order to address an accurate LC-MS quantitation of low-abundance *N*-glycopeptide structures, the MS sensitivity needs to be enhanced and the LC separation improved. The current strategies to increase glycopeptide sensitivity use either enrichment or fractionation methods [15,16,17]. Examples include the immunoprecipitation of glycoproteins from complex serum and plasma samples, and the separation of sialylated glycopeptides, based on their structural differences, using HILIC or lectin enrichment approaches [18,19,20,21].

An MS-based approach that can provide enhanced sensitivity is multiple reaction monitoring (MRM), which increases the glycopeptide detection by scanning the specific ions that represent the target structures. MRM increases the analyte detection sensitivity, but is limited by its inability to perform MS^n^ identification of the scanned ions, which is an important drawback in the evaluation of complex biological samples [22,23]. Alternatively, parallel reaction monitoring (PRM) is a promising strategy for identifying and quantifying specific glycopeptides in complex biological samples. This technique is possible due to modern highly sensitive mass analyzers, such as the Orbitrap and time-of-flight (TOF), which are capable of collecting the full MS^n^ spectra of the targeted ions, where the analytes can be accurately identified based on their *m*/*z* ratio and fragmentation patterns, and quantified using their most abundant and constant fragments [16,22]. For PRM quantitation, the fragmentation of the targeted ion is a relevant parameter that needs to be accurately evaluated, as most fragmentation mechanisms affect the glycan and peptide moieties of the glycopeptide differently. A soft fragmentation strategy, such as collision-induced dissociation (CID), provides high abundance and constant glycopeptide B and Y ions from the glycan portion, commonly glycan fragments without peptide information [24]. In contrast, high-energy collision dissociation (HCD) and electron transfer/higher-energy collision dissociation (EThcD) strategies generate glycan and peptide fragments, which allows for complete identification of the glycopeptide structures [10,17,25].

In this study, we integrated two strategies to evaluate the microheterogeneity of serum haptoglobin, focusing on the glycosylation sites Asn184, Asn207, and Asn241. Haptoglobin was extracted from serum samples of patients with NASH cirrhosis and NASH-related HCC. The first strategy involved the use of a long nano-C18 column (50 cm), to ensure a wide separation of the tryptic and Glu-C digested haptoglobin isomeric *N*-glycopeptides. The second strategy was based on a MS-PRM approach, directed to enhance the detection sensitivity of important low-abundance sialylated and fucosylated glycopeptides that change in abundance during the development of HCC [14,26]. We were able to identify site-specific glycosylation changes in serum haptoglobin that was extracted from patients with NASH cirrhosis and NASH-related HCC, where these changes were strongly related to the development of *N*-glycopeptide isomeric structures. Of significance, the statistical analysis of the data found seven haptoglobin *N*-glycopeptides that can be used alone, combined with AFP, or combined themselves, to better differentiate between cirrhosis and HCC group samples than previous approaches.

## 2. Results and Discission

### 2.1. LC-PRM-MS Strategy

**Figure 1:**
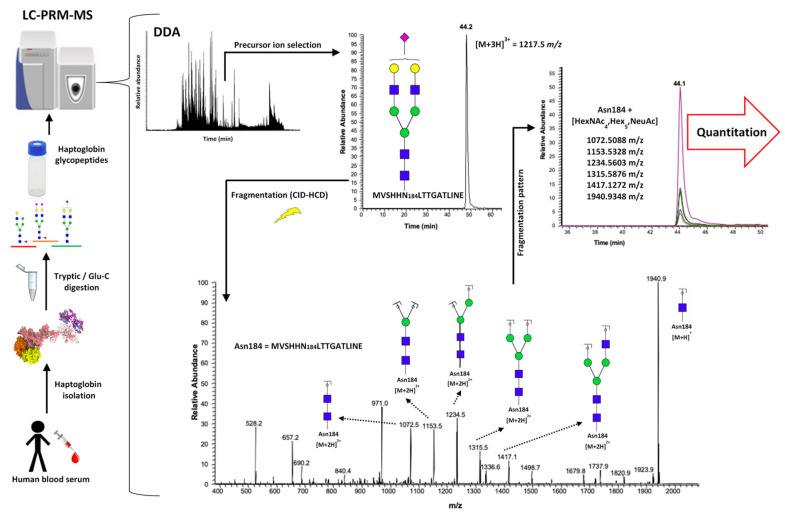
Workflow for the parallel reaction monitoring mass spectrometry (PRM-MS) analysis of serum haptoglobin *N*-glycopeptides.

A targeted PRM approach was applied for the quantitation of the intact *N*-glycopeptides of serum haptoglobin, to characterize the alterations in site-specific glycopeptide forms between cirrhosis and HCC. The experimental workflow is shown in Figure 1. Haptoglobin was purified from a 20 μL aliquot of serum from each patient, by using an HPLC-based antibody-immobilized column [1,10].

Serum haptoglobin has four glycosylation sites (Asn184, Asn207, Asn211, and Asn241), which can be observed after a two-step enzymatic digestion, using trypsin and Glu-C [10]. In this work, we focused on the glycosylation sites Asn184, Asn207, and Asn241; where we were unable to identify any *N*-glycopeptides from the glycosylation site Asn211, in a reproducible number of samples, to apply reliable evaluation [10,12]. A pooled sample was analyzed in data-dependent acquisition (DDA) mode and used to identify the *N*-glycopeptides present in the samples, based on monoisotopic mass, charge, retention time, and MS^2^ spectra (Supplementary Table S1).

The specific information for each detected *N*-glycopeptide structure was targeted in the LC-MS-PRM approach on the Orbitrap QExactive (Thermo). The selections of the precursor ions for all the identified glycopeptide structures was based on the signal intensity, so that in each case, the most abundant ion was used in the PRM strategy. Three charged precursor ions were the common ionic glycopeptide species in the three evaluated glycosylation sites, except for the glycopeptides with tri- and tetra-antennary and mono-, di-, and tri-sialylated glycans attached to the glycosylation site Asn241; where these structures presented four charged ions (Supplementary Table S1). The energy level was tested in the HCD cell of the QExactive, before the application of the PRM strategy. The most appropriate collision energy, to produce stable and abundant oxonium and core Y fragment ions of the glycan portion, was 25 eV. Additionally, the fragmentation that was produced allowed us to confirm the site-specific glycosylation, by the observation of abundant Y1 ions with *m*/*z* values of 1940.9333, 1176.5484, and 1998.0920, for the sites Asn184, Asn207, and Asn241, respectively. The backbone peptide fragments were observed in low abundance in the three glycosylation sites (Supplementary Figure S1). Six of the most representative and abundant fragment ions were selected for each *N*-glycopeptide, and their peak area was calculated using Xcalibur (Thermo) software (Supplementary Table S1). After the area under the curve was computed, the data were normalized for each glycosylation site based on the total area of all the site-identified glycopeptides and their calculated relative abundance. Significant differences in abundance, between the cirrhosis and HCC samples, were calculated using the t-test for each glycosylation site. Any *p*-values < 0.05 were considered to be a significant change; Supplementary Table S2 shows the data obtained for the cirrhosis and HCC samples. The retention time of the evaluated structures was such that the *N*-glycopeptides for the glycosylation site Asn184, eluted between 42 and 54 min, for the Asn207 site between 40 and 60 min, and for the Asn241 site between 61 and 88 min. For all the sites, a retention time pattern, based on the glycan moiety, was observed (Supplementary Table S1). For all the glycosylation sites, the first glycopeptides to be eluted were the structures with small biantennary mono- and di-sialylated glycans attached. The *N*-glycopeptides with sialylated fucosylated glycans attached eluted according to the number of sialic acid molecules that presented in the glycan moiety, regardless of the antennae number (Supplementary Table S1). The core or branch fucosylation of important *N*-glycopeptides was performed by the evaluation of their fragmentation, and an example of this can be found in Supplementary Figure S2. A pooled sample was used to determine the sialic acid linkage of some important structures, by the application of α2,3 neuraminidase enzyme digestion; a description can be found in Supplementary Figure S3. To facilitate the glycopeptide description, the nomenclature used is as follows. For glycans, a four-digit nomenclature represents the number of HexNAc, Hex, Fuc, and Neu5Ac molecules that can also be expressed with four digits “1-1-1-1” (*N*-acetylhexosamine, hexose, fucose, and *N*-acetylneuraminic acid, respectively). The peptide moiety was described as MVSHHN184LTTGATLINE = Asn184, NLFLN207HSE = Asn207, and VVLHPN241YSQVDIGLIK = Asn241.

**Figure 2:**
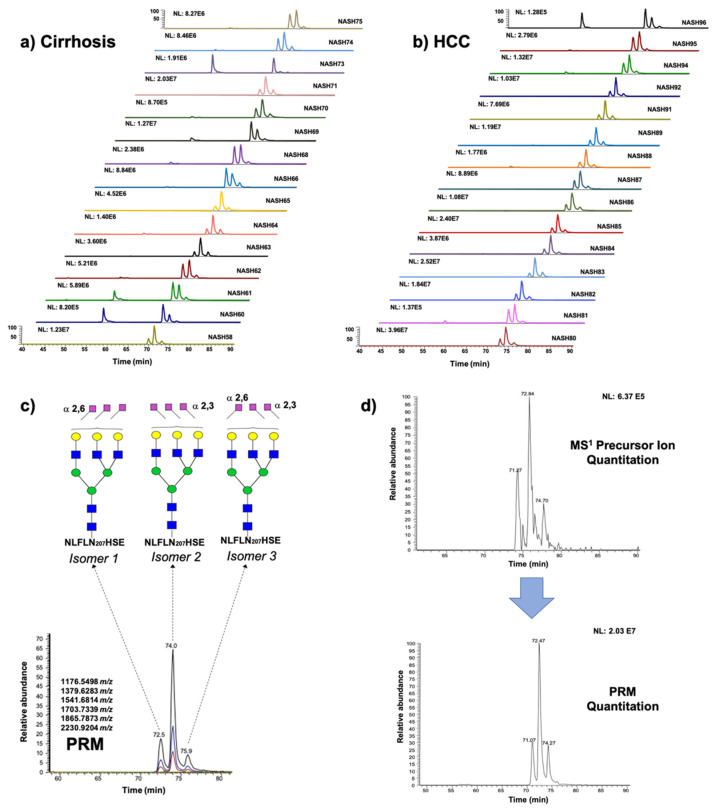
EICs for the glycopeptide structure NLFLN207HSE + HexNAc_5_Hex_6_Neu5Ac_3_ derived from (**a**) cirrhosis and (**b**) hepatocellular carcinoma patients; (**c**) PRM quantitation of the glycopeptide structure and sialic acid linkage of the isomeric glycan moieties; (**d**) EICs for the glycopeptide structure NLFLN207HSE + HexNAc_5_Hex_6_Neu5Ac_3_, 1278.8295 *m*/*z*. Sensitivity difference between full-scan MS ion extraction and PRM approach.

Figure 2 describes the data treatment, using the glycopeptide structure NLFLN207HSE + HexNAc_5_Hex_6_Neu5Ac_3_ as an example. Figure 2a,b depict the extracted ion chromatograms (EICs) for the abovementioned glycopeptide that was derived from all the cirrhosis and HCC patients, respectively. The corresponding precursor ion for the glycopeptide structure has a triple-charge *m*/*z* value of 1181.7977, it eluted in a retention time of about 72 min, and it produced monocharged fragment ions, principally of the core glycan portion (the *m*/*z* values of the targeted fragment ions were 1176.5498, 1379.6283, 1541.6814, 1703.7339, 1865.7873, and 2230.9204 for the PRM quantitation). Figure 2c shows the capability of our strategy to resolve three isomeric structures. Representative TICs of the isomeric structures that were observed in the samples are described in Supplementary Figure S4. Figure 2d shows the sensitivity enhancement of our PRM approach versus the full-scan MS signal observed in the pooled sample that was injected with the same concentration. This increase in sensitivity allowed us to address an accurate quantitation of low-abundant sialylated fucosylated glycopeptide structures that have known importance in HCC detection [27].

### 2.2. Haptoglobin Microheterogeneity

Based on our early research efforts, as described by Huang et al. and Zhu et al., the isomeric glycan profile of serum haptoglobin, and its glycosylation site heterogeneity, were determined [1,10]. These works demonstrated the importance of the isomeric structures, either glycan or glycopeptides, in the differentiation of patients with NASH cirrhosis and NASH-related HCC. Huang et al. reported seven sialylated fucosylated isomeric glycans, with the structures HexNAc_4_Hex_5_FucNeuAc, HexNAc_5_Hex_6_FucNeuAc_3_, and HexNAc_5_Hex_6_Fuc_2_NeuAc_3_. All of these structures showed significant changes in abundance between the two disease states [1]. In a related work, Jin et al. found 12 isomeric glycans, with significant differences between healthy and HCC patients [14]. The development of an LC strategy that allows the separation of isomeric *N*-glycopeptides was addressed, based on the use of a long C18 column (50 cm) and high temperature. The strategy was adopted from the work of Ji et al., where they reported the separation of isomeric *O*- and *N*-glycopeptides from tryptic-digested α1-acid glycoprotein (AGP), and evaluated the effects of temperature on the isomeric separation as well [28].

We were able to accurately identify and quantify a total of 73 isomeric structures that corresponded to 42 *N*-glycopeptide forms from the tryptic/Glu-C-digested haptoglobin, as shown in Table 1. The distribution of the *N*-glycopeptides in the glycosylation sites were as follows: 13 isomeric structures that corresponded to 11 *N*-glycopeptide forms in the glycosylation site Asn184; 44 isomeric structures that corresponded to 19 *N*-glycopeptide forms in the glycosylation site Asn207; and 16 isomeric structures that corresponded to 12 *N*-glycopeptide forms in the glycosylation site Asn241. The glycosylation site Asn207 was found to have the most abundant diversity of *N*-glycopeptides, as well as the most abundant isomeric *N*-glycopeptide structures.

**Figure 3:**
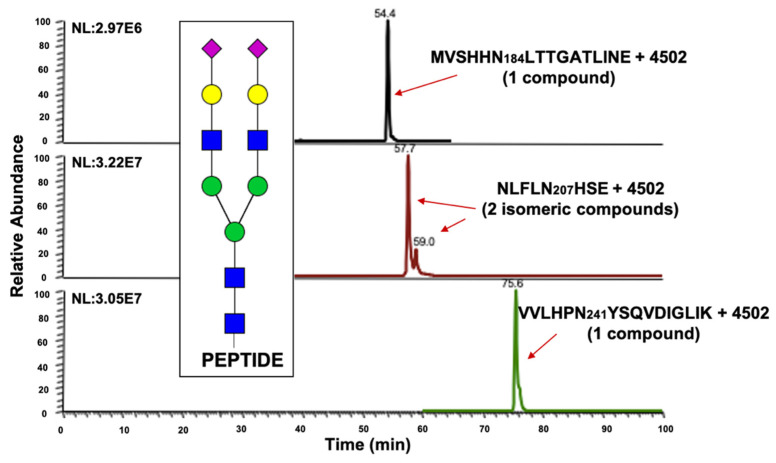
*N*-glycan distribution among glycosylation sites of the glycan structure HexNAc_4_Hex_5_Neu5Ac_2_ (4-5-0-2). Glycan nomenclature: HexNAc, Hex, Fuc, NeuAc (*N*-acetylhexosamine, hexose, fucose, *N*-acetylneuraminic acid, respectively).

We found 7 common glycan structures between the three glycosylation sites Asn184, Asn207, and Asn241; 10 between the sites Asn184 and Asn207; 11 between the sites Asn207 and Asn241; and 7 between the sites Asn184 and Asn241 (Table 1). The glycan structure HexNAc_4_Hex_6_Neu5Ac was only observed in the glycosylation site Asn184; the structure HexNAc_3_Hex_4_Neu5Ac was only observed in the site Asn207; and the structure HexNAc_6_ Hex_7_Neu5Ac_3_ was observed only in the site Asn241, which matched with the findings of Zhu et al. [10]. In addition, some of the common structures showed a different number of isomeric peaks among the glycosylation sites. For example, the glycan composition HexNAc_4_Hex_5_Neu5Ac_2_ (4-5-0-2) was present in the three sites, but, interestingly, two isomeric glycans were observed only in the site Asn207 (Figure 3). Similar results were observed for the other common glycan structures among the three glycosylation sites (Table 1). The results demonstrated the ability of our analytical strategy to unravel the glycan microheterogeneity of the glycosylation site NLFLN207HSE, and clearly described the distribution of several isomeric glycan structures among the three evaluated haptoglobin glycosylation sites. According to previous research, 35 of the 42 identified *N*-glycopeptides in our research were common with the reported work of Zhu et al., who evaluated similar analytical samples with an LC-EThcD-MS^2^ analytical strategy and found a large number of *N*-glycopeptides; however, their work did not report isomeric structures [10].

**Figure 4:**
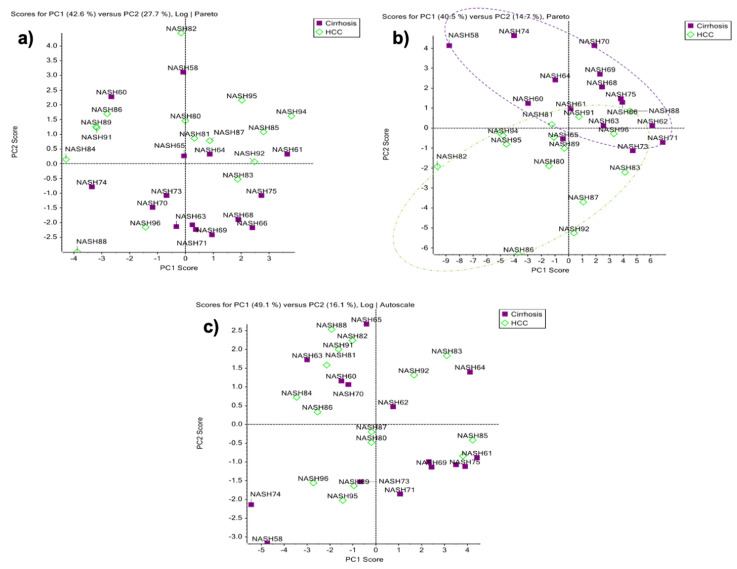
PCA plots for the glycosylation sites derived from human serum haptoglobin from patients with cirrhosis and HCC. (**a**) MVSHHN184LTTGATLINE; (**b**) NLFLN207HSE; and (**c**) VVLHPN241YSQVDIGLIK.

Principal component analysis (PCA) was performed, to evaluate the ability of the obtained data to differentiate between cirrhosis and HCC. The analysis was performed with the MarkerView^®^ (AB Sciex) software, using the normalized data for each glycosylation site (Figure 4). The PCA plots of the sites MVSHHN184LTTGATLINE (Figure 4a) and VVLHPN241YSQVDIGLIK (Figure 4c) showed that the obtained data were not able to separate the disease cohorts. In comparison, the PCA analysis for the glycosylation site NLFLN207HSE showed an important separation between the cirrhosis and HCC sample groups. As discussed previously, the site Asn207 has an important number of isomeric *N*-glycopeptides, suggesting that the differentiation of the two cohorts is possible due to the microheterogeneous development of the glycosylation site (Figure 4b). The closest points between two cohorts corresponds to samples in the early HCC stage and the cirrhosis samples with the highest AFP (alpha-fetoprotein) levels, which is glycoprotein used for the detection of HCC (Supplementary Table S3). Additionally, the separation of the sample groups was evaluated by gender (Supplementary Figure S5), where the PCA plots of the glycosylation site NLFLN207HSE showed that the obtained data can differentiate between cirrhosis and HCC in the female cohort (Supplementary Figure S5a), and in the male cohort as well (Supplementary Figure S5b).

**Figure 5:**
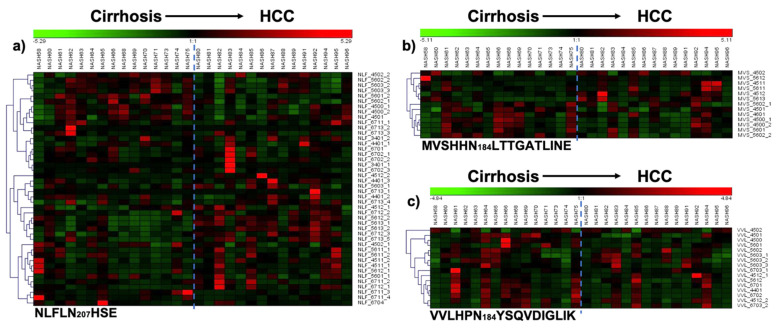
Heat maps for glycosylation site. (**a**) NLFLN207HSE (NFL + “Glycan”); (**b**) MVSHHN184LTTGATLINE (MVS + “Glycan”); and (**c**) VVLHPN241YSQVDIGLIK (VVL + “Glycan”). Glycan nomenclature: HexNAc, Hex, Fuc, NeuAc (*N*-acetylhexosamine, hexose, fucose, *N*-acetylneuraminic acid, respectively). Color red (up-regulation) and color green (down-regulation).

Site-specific heat maps were obtained for each of the following glycosylation sites: Asn184, Asn207, and Asn241 (Figure 5), and used to evaluate the site glycome differences between the cirrhosis and HCC samples. For the site MVSHHN184LTTGATLINE, the heat map allows us to identify two principal changes. The bi- and tri-antennary sialylated glycans 4-5-0-2, 5-6-0-1, 5-6-0-2, and 5-6-0-3, were more abundant in the HCC than in the cirrhosis samples. Otherwise, the bi- and tri-antennary sialylated glycans 3-4-0-1, 4-4-0-1, 6-7-0-1, and 6-7-0-2, were more abundant in the cirrhosis than in the HCC samples (Figure 5b).

The isomeric separation that was achieved by our analytical strategy increased the number of glycopeptide structures in the glycosylation site NLFLN207HSE, and thus its structural information (microheterogeneity), Figure 5a. The heat map for this site describes three different groups of glycans, with important changes in abundance between the cirrhosis and HCC cohorts. Sialylated glycans had different abundance patterns in this site; the tri-antennary mono-, di-, and tri-sialylated structures 5-6-0-1, 5-6-0-2, and 5-6-0-3, were more abundant in the cirrhosis than the HCC samples. However, the mono-, di-, and tetra-antennary mono- and di-sialylated glycans 3-4-0-1, 4-4-0-1, 6-7-0-1, and 6-7-0-2, were more abundant in the HCC than the cirrhosis samples. In the case of sialylated fucosylated glycans, the heat map showed similar changes in abundance for almost all the structures; these glycans were in low abundance in the cirrhosis samples, and increased considerably for the HCC samples, as expected from prior works [1,2,12]. The heat map for the glycosylation site VVLHPN_241_YSQVDIGLIK showed changes in abundance for the glycan structures 4-5-0-1 and 4-5-0-2; these glycans were more abundant in the cirrhosis than the HCC samples, Figure 5c.

**Figure 6:**
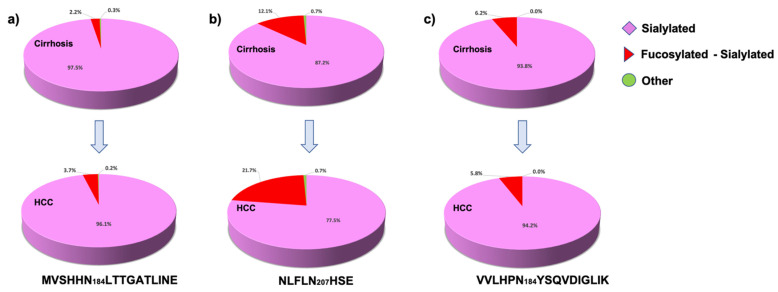
Glycome changes in abundance of serum haptoglobin associated with cirrhosis and HCC samples. Serum haptoglobin sites, as follows: (**a**) MVSHHN184LTTGATLINE; (**b**) NLFLN207HSE; and (**c**) VVLHPN241YSQVDIGLIK. The significant changes were observed on the glycosylation site NLFLN207HSE, where the sialylated glycans changed from 87.2% to 77.5% (*p*-value 0.0001), and the fucosylated sialylated glycans changed from 12.1% to 21.7% (*p*-value 0.0001).

The site-specific glycome changes associated with group-glycan types was also evaluated, using pie graphs. For comparison, the data were separated into the following three main glycan groups: sialylated, sialylated fucosylated, and other structures (Figure 6). As expected, according to the PCA plots and heat map analyses, the glycome for the sites MVSHHN184LTTGATLINE and VVLHPN_241_YSQVDIGLIK, did not show significant changes in abundance for sialylated and sialylated fucosylated glycans, Figure 6a,c, respectively. For the site NLFLN207HSE, we observed relative abundance values of 87.2% and 12.1%, for the sialylated and sialylated fucosylated glycans, respectively, in the cirrhosis samples. In comparison, the relative abundance values of the sialylated and sialylated fucosylated glycopeptides changed for the HCC samples, to 77.5% and 21.7% respectively (Figure 6b). According to the results observed in the pie graphs, only the glycome of the haptoglobin glycosylation site NLFLN207HSE showed significant changes between the two sample groups. Among the observed changes, the high abundance of sialylated glycans presented in the cirrhosis samples decreased by around 9.7% in HCC, and the sialylated fucosylated glycans increased by 9.6% from cirrhosis to HCC.

The results observed in the PCA plots, heat maps, and pie graphs showed consistent glycosylation differences in serum haptoglobin between the cirrhosis and HCC samples. In addition, the important number of isomeric *N*-glycopeptides resolved by our LC-PRM-MS strategy, upgrade the differentiation between the evaluated sample groups. Most of the changes were observed in the glycosylation site NLFLN207HSE, which is information that elucidated the position of the protein glycosylation differences between the cirrhosis and HCC samples.

### 2.3. Differentially Relative Abundances of Haptoglobin N-Glycopeptides in Cirrhosis and HCC

**Figure 7:**
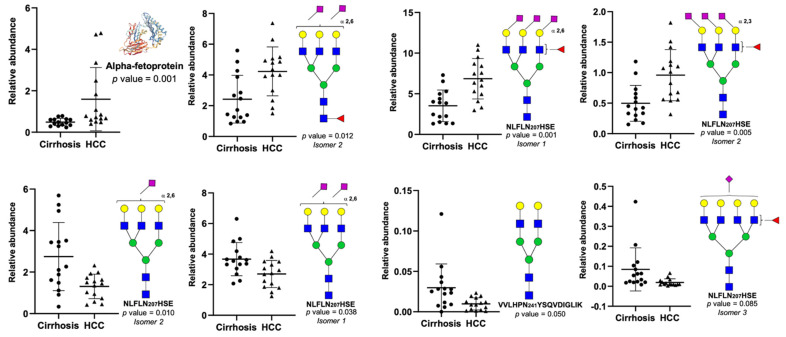
Alpha-fetoprotein and glycopeptide structures with significant difference between cirrhosis and HCC samples. NLFLN207HSE (Asn207), and VVLHPN241YSQVDIGLIK (Asn241).

Since our LC-PRM-MS approach provided clear differences in site-specific *N*-glycopeptides between cirrhosis and HCC, we further focused on investigating the differentially relative abundances of *N*-glycopeptides between both diseases. Initially, seventeen site-specific *N*-glycopeptides were found to have the most significant differences in abundance between cirrhosis and HCC, Supplementary Table S4. Further statistical analyses were applied to these glycopeptides, in order to avoid possible gender bias, due the large number of female samples presented in the cirrhosis cohort. The *N*-glycopeptide significance was evaluated by adjusting both the sample cohorts to the same gender ratio. Three sample sets were tested; Supplementary Table S5 shows the complete evaluation to designate the significant *N*-glycopeptides. The large number of parameters used in this evaluation can increase the probability that the observed results were accidental. Therefore, “Bonferroni correction” was applied to the observed *p*-values. The corrected *p*-values can be observed in Supplementary Table S5. By using this evaluation, we intended to increase the accuracy of the presented results. Four site-specific *N*-glycopeptides were found to have significant differences in abundance between cirrhosis and HCC, despite the gender factor. All of them were found in the glycosylation site NLFLN207HSE. The significant *N*-glycopeptides observed were as follows: the tri-antennary sialylated isomeric glycopeptides Asn207 + 5-6-0-1 isomer 2 and the Asn207 + 5-6-0-2 isomer 1; and the sialylated fucosylated isomeric glycopeptides Asn207 + 5-6-1-3 isomers 1 and 2. The statistical comparation of these *N*-glycopeptides between gender groups showed that no significant bias was introduced by gender, Supplementary Table S6. Additionally, the PCA plots of the glycosylation site NLFLN207HSE showed that the obtained data differentiated between cirrhosis and HCC in the female and male cohorts (Supplementary Figure S5a–c). Figure 7 shows the dispersion plots, *p*-values, and cartoons of the *N*-glycopeptides with the most significant changes between the cirrhosis and HCC samples. Among these structures, the isomeric *N*-glycopeptide Asn207 + 5-6-1-3 showed the best capabilities differentiating between both the sample groups, with a *p*-value of 0.001 for the isomeric form one and a *p*-value of 0.005 for the isomeric form two, and when the glycopeptide was processed as a single structure, the *p*-value was 0.001. Furthermore, we previously reported the significance of the glycan structure “5-6-1-3” in our haptoglobin glycomics studies of HCC [1]. The *p*-values for other *N*-glycopeptides with significant changes were 0.001 and 0.038 for the glycopeptides Asn207 + 5-6-0-1 isomer 2 and Asn207 + 5-6-0-2 isomer 1, respectively. Our findings strongly correlated with the reported glycosylation alterations of serum haptoglobin in hepatic cancer. Ang et al. reported the increase in glycan fucosylation, the decrease in sialylated glycans, and the increase in α2,6 sialic acid linkage [29], these changes correlated with the alterations observed in the *N*-glycopeptides and the significant changes described between cirrhosis and HCC by our analytical strategy, Figure 7. Four of the glycopeptides that are depicted in Figure 7 have fucose on their glycan structures and an increase in abundance in HCC (Asn207 + 5-6-1-2, Asn207 + 5-6-1-3, and Asn207 + 6-7-1-1), and two glycopeptides with α2,6 sialylation that have a decrease in abundance in HCC (Asn207 + 5-6-0-1, and Asn207 + 5-6-0-2). Shu et al. described the increase in glycans with type Le^x^ fucosylation [30], which is a characteristic that can be observed for the isomeric *N*-glycopeptides Asn207 + 5-6-1-3 and Asn207 + 6-7-1-1, Figure 7. Zhu et al. reported the increase in branching tri-, and tetra-antennary glycans [31], which are glycan structures that are observed in six of the depicted *N*-glycopeptides with significant changes, Figure 7. Additionally, our overall results agree with the extensively reported increase in fucosylation [32,33,34,35].

Additionally, to assess the ability of the listed haptoglobin *N*-glycopeptides to differentiate between cirrhosis and early HCC, the relative abundance of the glycopeptides was compared between the cirrhosis and HCC TNM 1 samples (Supplementary Tables S3 and S4). To avoid gender bias in the comparison, the ratio of both the sample groups was the same. Three sample sets were tested; Supplementary Table S7 shows the complete evaluation to designate the significant *N*-glycopeptides that are capable of differentiating between cirrhosis and early HCC. The large number of parameters used in this evaluation can increase the probability that the observed results are accidental. Therefore, “Bonferroni correction” was applied to the observed *p*-values. The corrected *p*-values can be observed in Supplementary Table S7. The site-specific *N*-glycopeptide NLFLN207HSE + 5-6-0-1 isomer 2 was found to have significant differences in abundance between cirrhosis and early HCC, with a *p*-value of 0.006. Otherwise, the *p*-value of the same set of samples that were obtained for AFP, was 0.354 (Supplementary Tables S4 and S7). AFP is currently used in the detection of HCC, where the levels of this glycoprotein increase considerably in the late stages of HCC. Unfortunately, AFP does not have sufficient sensitivity and specificity to differentiate between liver cirrhosis and early HCC [7]. Interestingly, the non-fucosylated *N*-glycopeptide Asn207 + 5-6-0-1, better differentiated between the cirrhosis and early HCC group samples than AFP.

The receiver operating characteristic curve (ROC) is a graphical plot that illustrates the diagnostic ability of a binary classifier system, such as two related diseases or two stages in the progression of a particular disease. Thus, we evaluate the performance of the isomeric *N*-glycopeptides that were deciphered by our LC-PRM-MS strategy, using the area under the curve (AUC) values of their corresponding ROC curves to describe the cirrhosis and HCC relative abundance differences (Table 2). Initially, the AUC values observed for the haptoglobin *N*-glycopeptides were compared against AFP. The results showed equal AUC values of 0.85 for AFP and for the *N*-glycopeptide Asn207 + 5-6-1-3 isomer 1, Table 2. The ROC curves were performed for AFP and the *N*-glycopeptide Asn207 + 5-6-0-1 isomer 2, using early HCC samples, as was explained above. The AUC value for AFP was 0.81, otherwise, the haptoglobin *N*-glycopeptide NLFLN207HSE + 5-6-0-1 isomer 2 showed better performance than AFP, with an AUC value of 0.88 (Table 2).

The site-specific *N*-glycopeptide analyses have the advantage that different structures observed in a single assay can be combined, to gain sensitivity and specificity in the differentiation of two sets of samples. Therefore, we evaluated different glycopeptides as complement of AFP and different groups of glycopeptides that showed common changes between the cirrhosis and HCC samples (Table 2). The results showed seven combinations with higher AUC values than AFP alone. Within them, the combination of AFP and the two isomeric *N*-glycopeptides Asn207+ 5-6-1-2 isomer 2, and Asn207 + 5-6-1-3 isomers 1 and 2, showed an AUC value of 0.98. This is a higher AUC value than the 0.83 and 0.84 previously reported by Zhu et al. [31], and Asazawa et al. [35], respectively, in a similar evaluation. The combination of AFP with other glycopeptides showed the next results. AFP + (Asn207 + 5-6-1-2 isomer 2) with an AUC of 0.88, AFP + (Asn207 + 5-6-1-3 isomer 1) with an AUC of 0.94, and AFP + (Asn207 + 5-6-1-3 isomer 2) with an AUC of 0.93. The combination of the two glycopeptides Asn207 + 5-6-1-2 isomer 2 and Asn207 + 5-6-1-3 isomer 2 resulted with an AUC of 0.87. The combination of all the significant sialylated fucosylated glycopeptides in the glycosylation site Asn207, resulted with an AUC of 0.91, and the combination of all the significant sialylated glycopeptides from the glycosylation sites Asn207 and Asn184, resulted with an AUC of 0.91 (Table 2). The results demonstrated that the combination of *N*-glycopeptides, either with AFP or between themselves, considerably enhanced the accuracy of the differentiation of the cirrhosis and the HCC sample groups. The combination of AFP with fucosylated sialylated *N*-glycopeptides showed better results, because both the analytes increased their abundance from cirrhosis to HCC. Unlike AFP, the sialylated *N*-glycopeptides decreased in abundance from cirrhosis to HCC. The sialylated *N*-glycopeptides were the most abundant structures that were quantified in our analytical strategy, but, unfortunately, the high variability of this type of glycopeptide in HCC samples limited their application in the differentiation of cirrhosis and HCC; Supplementary Table S4 shows the standard deviation differences between the two sample groups. The statistical comparation of these *N*-glycopeptides between the gender groups showed that no significant bias was introduced by gender, Supplementary Table S6.

The GALAD score is a serum biomarker that predicts the probability of having HCC in patients with chronic liver diseases. Best et al. used the GALAD score to differentiate between NASH control and NASH HCC, for both the cases with and without cirrhosis. When data from NASH cirrhosis was compared with NASH HCC, they observed an AUC value of 0.93, which is lower than the 0.98 that we observe with our strategy. For the comparison of NASH cirrhosis and NASH early HCC, they observed an AUC value of 0.85, which is lower than the 0.88 that was observed with our strategy. The des-gamma-carboxy prothrombin (DCP) is another HCC biomarker that was also evaluated in the same cohorts, and the AUC values were lower than the values observed using the GALAD score, and lower than those observed in our strategy [36].

Although the investigation presented here was based on a small sample number in the comparative cohort. The principal outcome of our LC-PRM-MS approach intended to elucidate a strategy that is capable of robustly identifying site-specific changes in glycoprotein-related diseases. Under this strategy, we were able to observe that several changes in protein glycosylation happen at the micro level, such as the glycan isomerism. Additionally, the analytical strategy used provided a high certainty in the identification of relatively small biomolecules present in complex biological samples. Precise and accurate identification can be achieved using the fingerprint mass spectra of the target molecules, which is an important advantage, due the lack of standards in bioanalysis. Regardless of the sample size, a proof of the consistent results is the strong correlation of our data with previous studies. Moreover, we incorporated additional supporting parameters that increased AFP sensitivity and specificity, even in the early HCC stages. As was mentioned earlier, the application of a PRM approach requires expensive and highly accurate LC-MS systems, which is not necessarily a disadvantage, because the discovery of biomarker molecules that are capable to use in disease diagnosis is of high importance for human health. Besides, after the discovery process is concluded, the analysis of single or groups of biomolecules can be easily transferred to a common and accessible analytical technique, such as HPLC.

## 3. Materials and Methods

### 3.1. Materials

Trypsin/Lys-C mix mass spectrometry grade and Glu-C sequencing grade were purchased from Promega (Madison, WI, USA). The anti-human haptoglobin antibody was purchased from Abcam (Cambridge, MA, USA). Acetonitrile, methanol, and mass spectrometry-grade water were purchased from Fisher Scientific (Fair Lawn, NJ, USA). Serum samples were provided by the University of Michigan and the University of Texas Southwestern Hospital according to IRB approval; 15 cases of liver NASH cirrhosis, and 15 cases of NASH-related HCC. The clinical information associated with the samples used in this study is summarized in Table 3. The patients were diagnosed by imaging and biopsy. All the HCC patients in the study had cirrhosis, which is the intended population for HCC screening. Early HCC was identified as a single tumor ≤2 in diameter according to the BCLC staging system, the complete clinical information can be observed in Supplementary Table S3.

### 3.2. Purification of Haptoglobin from Human Serum

Haptoglobin was purified from a 20 μL aliquot of serum for each patient using an HPLC-based antibody-immobilized method developed in house as previously reported [1,10]. Before enzymatic digestion, the purity of the eluted haptoglobin was confirmed by 1D SDS-PAGE followed by silver staining using the ProteoSilverTM Plus silver stain kit (Sigma).

### 3.3. Tryptic and Glu-C Haptoglobin Digestion

The purified serum haptoglobin was resuspended with 20 μL of 50 mM NH_4_HCO_3_ and denatured at 90 °C in a water bath for 15 min. The denatured glycoprotein was reduced by the addition of 0.5 μL of 200 mM dithiothreitol (DTT) and incubation at 60 °C for 45 min. Then, the glycoprotein was alkylated by the addition of 2.0 μL of 200 mM iodoacetamide (IAA) and incubation at 37 °C for 45 min. A second addition of 0.5 μL of 200 mM DTT and incubation at 37 °C for 30 min was performed to quench the IAA excess. Trypsin was added to the treated glycoprotein sample at a concentration ratio of 1:25 and incubated at 37 °C overnight. After incubation, the digestion was quenched at 90 °C for 15 min. In a second enzymatic digestion, Glu-C was added to the tryptic-digested sample at a concentration ratio of 1:25 and incubated at 37 °C overnight. The sample was finally dried down in a SpeedVac concentrator.

### 3.4. LC-PRM-MS Analysis

The dried tryptic and Glu-C digests were resuspended in a solution of 2% acetonitrile (MeCN), 0.1% formic acid (FA). Five microliters of the reconstituted sample were injected onto a C18 trap column (75 μm × 10 cm, 2 μm, 100 Å; Thermo Scientific, Pittsburgh, PA, USA) for 10 min, and the samples were then transferred to an Aclaim PepMap C18 capillary column (75 μm × 50 cm, 2 μm, 100 Å; Thermo Scientific, Pittsburgh, PA, USA) using an Ultimate 3000 nanoUHPLC system (Dionex, Sunnyvale, CA, USA). The flow rate was set to 300 nL/min with a temperature of 60 °C. Mobile phase A was an aqueous mixture with 2% of MeCN, and 0.1% of FA, while mobile phase B was a mixture of MeCN with 0.1% of FA. The analytical gradient was 100 min long, and started at 2% mobile phase B for the initial 10 min, then increased to 38% at 11 min. During the next 70 min the organic phase B gradually developed to 60%. Subsequently, it ramped up to 90% in a period of 3 min and was maintained for 4 min. Finally, the percentage of organic phase B dropped to 2% in 1 min and was kept at that condition to pre-equilibrate the system. The nanoUHPLC system was interfaced to a Q Exactive (Thermo Scientific, San Jose, CA, USA), and operated in positive ion mode for PRM-MS analysis. The fragmentation pattern of the precursor ions was evaluated with different collision energy (CE) levels and established as 25 eV. Then, a MS full-scan range of 300–2000 *m*/*z* and a MS^2^ scan range of 300–3000 *m*/*z* were applied to a pooled sample for the identification of the precursor ions, their fragments and retention times. All the identified precursor ions were included in the PRM-MS method with a retention time window of ±3 min, and a mass range of ±2 Da relative to the target mass. In order to compare the data from two cohorts, t test and Wilcoxon test were performed and *p*-values < 0.05 were considered significant, the *p*-values observed in the evaluated groups were corrected using Bonferroni correction (*n* = 3, α = 0.05). We obtained area under receiver operating characteristic curve (AUC) values, and heat maps using the SPSS^®^ version 27 (IBM) software. PCA plots were obtained using the software MarkerView^®^ version 1.3 (AB Sciex).

## 4. Conclusions

In this study, we revealed the site-specific microheterogeneity (isomeric composition) of serum haptoglobin, and demonstrated that the developed isomeric *N*-glycopeptides can be used to differentiate NASH cirrhosis and NASH-related HCC. This assay incorporated a target parallel reaction monitoring (PRM) approach, with a long C18 column that was capable of resolving important isomeric glycopeptide structures at a high temperature. We were able to accurately quantify 72 isomeric structures corresponding to 42 glycopeptide compositions, which were structures that were distributed among the haptoglobin glycosylation sites as follows: thirteen in the site Asn184, forty-four in the site Asn207, and sixteen in the site Asn241. The glycosylation site NLFLN207HSE (Asn207) showed the largest number of glycan structures, most of them with isomeric forms. The microheterogeneity of this site was strongly related with the data ability to differentiate between the studied diseases, as can be observed in the PCA and heat map analysis. Additionally, quantitative analysis revealed four *N*-glycopeptides with significant changes in abundance between the cirrhosis and HCC samples (*p* < 0.05), all of them located in the site Asn207. Two of the significant *N*-glycopeptides were sialylated fucosylated structures that increased in abundance from cirrhosis to HCC. Two of the significant *N*-glycopeptides were tri-antennary sialylated structures that decrease in abundance from cirrhosis to HCC. The AUC values of the ROC curves were used to compare the accuracy of single and groups of *N*-glycopeptides in the differentiation of cirrhosis and HCC diseases. The results showed different group combinations with higher AUC values than AFP alone. Additionally, the *N*-glycopeptide Asn207 + 5-6-0-1 better differentiated between cirrhosis and early HCC than AFP.

This study confirmed that site-specific glycoproteomic analysis is an important tool to evaluate serum haptoglobin changes between cirrhosis and HCC samples. Additionally, we showed that the unraveling of the haptoglobin isomeric *N*-glycopeptides contributed to the enhanced differentiation between both diseases. Moreover, by applying our LC-PRM-MS strategy, we were able to identify single and groups of haptoglobin *N*-glycopeptides that have potential to differentiate between patients with NASH cirrhosis and NASH-related HCC.

## Figures and Tables

**Table 1:** Serum haptoglobin microheterogeneity, *N*-glycopeptide structures by glycosylation site including isomeric forms. MVSHHN184LTTGATLINE = Asn184, NLFLN207HSE = Asn207, and VVLHPN241YSQVDIGLIK = Asn241. Glycan nomenclature; HexNAc, Hex, Fuc, NeuAc (*N*-acetylhexosamine, hexose, fucose, *N*-acetylneuraminic acid, respectively).

Glycopeptide Backbone	Asn184	Asn207	Asn241
*N*-glycosylation Microheterogeneity	---	3-4-0-1, 2 Isomers	---
	---	4-4-0-1, 3 Isomers	4-4-0-1
	---	4-5-0-0, 2 Isomers	4-5-0-0
	4-5-0-0, 2 Isomers	4-5-1-1, 2 Isomers	---
	4-5-1-1	4-5-1-2, 2 Isomers	4-5-1-2, 2 Isomers
	4-5-1-2	4-5-0-1	4-5-0-1
	4-5-0-1	4-5-0-2, 2 Isomers	4-5-0-2
	4-5-0-2	---	---
	4-6-0-1	5-6-1-1, 2 Isomers	---
	5-6-1-1	5-6-1-2, 2 Isomers	5-6-1-2
	5-6-1-2	5-6-1-3, 2 Isomers	---
	5-6-1-3	5-6-0-1, 2 Isomers	5-6-0-1
	5-6-0-1	5-6-0-2, 2 Isomers	5-6-0-2
	5-6-0-2, 2 Isomers	5-6-0-3, 3 Isomers	5-6-0-3, 3 Isomers
	---	6-7-1-1, 4 Isomers	---
	---	6-7-1-2, 3 Isomers	---
	---	6-7-1-3, 5 Isomers	---
	---	6-7-0-1	6-7-0-1
	---	6-7-0-2, 3 Isomers	6-7-0-2
	---	---	6-7-0-3, 2 Isomers

**Table 2:** Area under the curve (AUC) comparing single-glycopeptide models, and group-glycopeptide models for the evaluation of cirrhosis and HCC samples. MVSHHN184LTTGATLINE = Asn184, NLFLN207HSE = Asn207, VVLHPN241YSQVDIGLIK = Asn241, and AFP = alpha-fetoprotein. Glycan nomenclature: HexNAc, Hex, Fuc, NeuAc (*N*-acetylhexosamine, hexose, fucose, *N*-acetylneuraminic acid, respectively).

**Single-Glycopeptide Model, Differentiation of Cirrhosis and HCC**			
**Glycopeptide**	**AUC**	**CI ^1^ (Low)**	**CI ^1^ (High)**
AFP	0.85	0.72	0.97
Asn207 + 5-6-1-2 isomer 2	0.81	0.65	0.97
Asn207 + 5-6-1-3 isomer 1	0.85	0.72	0.99
Asn207 + 5-6-1-3 isomer 2	0.83	0.68	0.98
Asn207 + 5-6-0-1 isomer 2	0.77	0.59	0.95
Asn207 + 5-6-0-2 isomer 1	0.58	0.58	0.93
Asn207 + 6-7-1-1 isomer 3	0.81	0.66	0.97
Asn241 + 4-5-0-0	0.79	0.62	0.96
AFP “early HCC”	0.81	0.57	1.00
Asn207 + 5-6-0-1 isomer 2 “early HCC”	0.88	0.65	1.00
**Group-Glycopeptide Model, Differentiation of Cirrhosis and HCC**			
**Group of Glycopeptides**	**AUC**	**CI (Low)**	**CI (High)**
AFP + (Asn207 + 5-6-1-2 isomer 2)	0.88	0.75	1.00
AFP + (Asn207 + 5-6-1-3 isomer 1)	0.94	0.87	1.00
AFP + (Asn207 + 5-6-1-3 isomer 2)	0.93	0.85	0.99
AFP + (Asn207 + 5-6-1-2 isomer 1) + (Asn207 + 5-6-1-3 isomers 1 and 2)	0.98	0.93	1.00
(Asn207 + 5-6-1-2 isomer 2) + (Asn207 + 5-6-1-3 isomers 1 and 2)	0.87	0.74	0.99
Sialylated Fucosylated in Asn207	0.91	0.80	1.00
Sialylated Fucosylated in Asn207 + Sialylated Fucosylated in Asn184	0.91	0.79	1.00

^1^ Confidence interval (95%).

**Table 3:** Summary of patient clinical information.

Disease Diagnosis	Cirrhosis	HCC
Number	15 ^1^	15 ^2^
Etiology	NASH	NASH
Gender (M/F)	3/12	7/8
Age (mean ± SD)	60 ± 9	63 ± 9
AFP level ^3^ (median), ng/mL	2.9	6.0
ALT level ^3^ (mean ± SD)	38 ± 20	47 ± 27
AST level ^3^ (mean ± SD)	44 ± 22	71 ± 50
MELD ^4^ score (mean ± SD)	8 ± 1.6	12.4 ± 6.7
TNM stage, % (I *, II, III, IV)	NA	47 */0/40/13

^1^ Cirrhosis samples: NASH58, 60, 61, 62, 63, 64, 65, 66, 68, 69, 70, 71, 73, 74, 75. ^2^ HCC samples: NASH80 *, 81, 82, 83, 84, 85, 86, 87 *, 88 *, 89 *, 91, 92, 94 *, 95 *, 96 *. ^3^ AFP, ALT, and AST levels were provided by Division of Gastroenterology and Hepatology, University of Michigan and Division of Digestive and Liver Diseases, University of Texas Southwestern. ^4^ MELD: model for end-stage liver disease.

## Data Availability

The mass spectrometry raw data generated during the study are deposited and available in Proteome Xchange (http://www.Proteomecentral.proteomeexchange.org/cgi/GetDataset, accessed on 6 August 2021) with the dataset identifier PXD023719, username: reviewer_pxd023719@ebi.ac.uk, password: 5Ma7yiNs.

## Supplementary Materials

The following are available online at https://www.mdpi.com/article/10.3390/metabo11080563/s1, Figure S1: representative mass spectra of the three glycosylation sites, Figure S2: interpretation of fucosylated glycopeptides, Figure S3: sialic acid linkage of important and abundant haptoglobin *N*-glycopeptides, Figure S4: EICs showing the achieved isomeric separation of important haptoglobin *N*-glycopeptides, Figure S5: PCA plot of *N*-glycopeptides derived from human serum haptoglobin from patients with cirrhosis and HCC, Figure S6: receiving operating characteristic (ROC) curve for the glycopeptide structures with statistical significance between cirrhosis and HCC patients. Table S1: PRM information for each of the target haptoglobin *N*-glycopeptides, Table S2: peak area and normalized abundance of all the identified haptoglobin *N*-glycopeptides from patients with cirrhosis and hepatocellular carcinoma (HCC), Table S3: complete clinical information, Table S4: descriptive statistics of haptoglobin *N*-glycopeptides with important changes between the cirrhosis and HCC samples, Table S5: determination of haptoglobin *N*-glycopeptides with significative changes in abundance between cirrhosis and HCC, using same gender ratio female:male in both sample cohorts, Table S6: statistical comparison between gender groups, and Table S7: determination of haptoglobin *N*-glycopeptides with significative changes in abundance between cirrhosis and early HCC, using same gender ratio female:male in both sample cohorts.
