# Supplementary material for: PRM-MS Quantitative Analysis of Isomeric N-Glycopeptides Derived from Human Serum Haptoglobin of Patients with Cirrhosis and Hepatocellular Carcinoma
Source: Metabolites. 2021 Aug 23;11(8):563. doi: 10.3390/metabo11080563 (PMC8400780; doi:10.3390/metabo11080563)
Supplement: Supplementary file 1 [file metabolites-11-00563-s001.zip › metabolites-1265071-supplementary.pdf]

PRM-MS quantitative analysis of isomeric *N*-glycopeptides derived from human serum haptoglobin of patients with cirrhosis and hepatocellular carcinoma

Cristian D. Gutierrez Reyes[1ⱡ], Yifan Huang[1ⱡ], Mojgan Atashi [1], Jie Zhang[2], Jianhui Zhu[2], Suyu Liu[3], Neehar D. Parikh[4], Amit G. Singal[5], J. Dai[3], David M. Lubman[2] and Yehia Mechref[1*]

1. Department of Chemistry and Biochemistry, Texas Tech University, Lubbock, TX
2. Department of Surgery, The University of Michigan, Ann Arbor, MI 48109
3. Department of Biostatistics, University of Texas MD Anderson Cancer Center, Houston, TX 77030
4. Division of Gastroenterology and Hepatology, The University of Michigan, Ann Arbor, MI 48109
5. Division of Digestive and Liver Diseases, University of Texas Southwestern Medical Center, Dallas, TX 75390

ⱡ These authors contribute equally to this work

*Corresponding author
Department of Chemistry and Biochemistry
Texas Tech University
Lubbock, TX 79409-1061
Email: yehia.mechref@ttu.edu
Tel: 806-742-3059
Fax: 806-742-1289

Table of Contents:

Supplementary Figures:

Supplementary Figure S1. Representative mass spectra of the three glycosylation sites. **a**) MVSHHN$_{184}$LTTGATLINE = Asn184, **b**) NLFLN$_{207}$HSE = Asn207, and **c**) VVLHPN$_{241}$YSQVDIGLIK = Asn241. * Common fragment ion with different charge.

Supplementary Figure S2. Interpretation of fucosylated glycopeptides; **a**) *N*-glycopeptide NLFLN$_{207}$HSE + 4-5-1-2 isomers 1 and 2, **b**) *N*-glycopeptide NLFLN$_{207}$HSE + 6-7-1-1. NLFLN$_{207}$HSE = Asn207.

Supplementary Figure S3. Sialic acid linkage of important and abundant haptoglobin *N*-glycopeptides (NLFLN$_{207}$HSE = Asn207).

Supplementary Figure S4. EICs showing the achieved isomeric separation of important haptoglobin *N*-glycopeptides. MVSHHN$_{184}$LTTGATLINE = Asn184, NLFLN$_{207}$HSE = Asn207, and VVLHPN$_{241}$YSQVDIGLIK = Asn241.

Supplementary Figure S5. PCA plot of *N*-glycopeptides derived from human serum haptoglobin from patients with cirrhosis and HCC, glycosylation site NLFLN$_{207}$HSE. Gender evaluation, **a**) cirrhosis (female) “blue” vs. HCC (female) “red”, **b**) cirrhosis (male) “blue” vs. HCC (male) “red”.

Supplementary Figure S6. Receiver operating characteristic (ROC) curve for the glycopeptide structures with statistically significance between cirrhosis and HCC patients. MVSHHN$_{184}$LTTGATLINE = Asn184, NLFLN$_{207}$HSE = Asn207, and VVLHPN$_{241}$YSQVDIGLIK = Asn241. Glycan nomenclature: HexNAc, Hex, Fuc, NeuAc (*N*-acetylhexosamine, Hexose, Fucose, *N*-acetylneuraminic acid).

Supplementary Tables:

Supplementary Table S1. PRM information for each of the target haptoglobin *N*-glycopeptides.

Supplementary Table S2. Peak area and normalized abundance of all identified haptoglobin *N*-glycopeptides from patients with cirrhosis and hepatocellular carcinoma (HCC).

Supplementary Table S3. Complete clinical information.

Supplementary Table S4. Descriptive statistics of haptoglobin *N*-glycopeptides with important changes between cirrhosis and HCC samples. MVSHHN$_{184}$LTTGATLINE = Asn184, NLFLN$_{207}$HSE = Asn207, VVLHPN$_{241}$YSQVDIGLIK = Asn241, and AFP = alpha-fetoprotein. Glycan nomenclature: HexNAc, Hex, Fuc, NeuAc (*N*-acetylhexosamine, Hexose, Fucose, *N*-acetylneuraminic acid).

Supplementary Table S5. Determination of haptoglobin *N*-glycopeptides with significative changes in abundance between cirrhosis and HCC using same ratio female : male in both sample cohorts (p value <0.05). MVSHHN$_{184}$LTTGATLINE = Asn184, NLFLN$_{207}$HSE = Asn207, VVLHPN$_{241}$YSQVDIGLIK = Asn241, and AFP = alpha-fetoprotein. Glycan nomenclature: HexNAc, Hex, Fuc, NeuAc (*N*-acetylhexosamine, Hexose, Fucose, *N*-acetylneuraminic acid).

Supplementary Table S6. Statistical comparison between gender groups. **a)** Comparison of the statistically significant N-glycopeptides between gender groups. **b)** Multiple-variable model adding gender. Single and group glycopeptide models, differentiation of cirrhosis and HCC. **c)** The corresponding logistic regression model. NLFLN$_{207}$HSE = Asn207, VVLHPN$_{241}$YSQVDIGLIK = Asn241, and AFP = alpha-fetoprotein. Glycan nomenclature: HexNAc, Hex, Fuc, NeuAc (*N*-acetylhexosamine, Hexose, Fucose, *N*-acetylneuraminic acid).

Supplementary Table S7. Determination of haptoglobin *N*-glycopeptides with significative changes in abundance between cirrhosis and **early HCC** using same gender ratio female : male in both sample cohorts. MVSHHN$_{184}$LTTGATLINE = Asn184, NLFLN$_{207}$HSE = Asn207, VVLHPN$_{241}$YSQVDIGLIK = Asn241, and AFP = alpha-fetoprotein. Glycan nomenclature: HexNAc, Hex, Fuc, NeuAc (*N*-acetylhexosamine, Hexose, Fucose, *N*-acetylneuraminic acid).

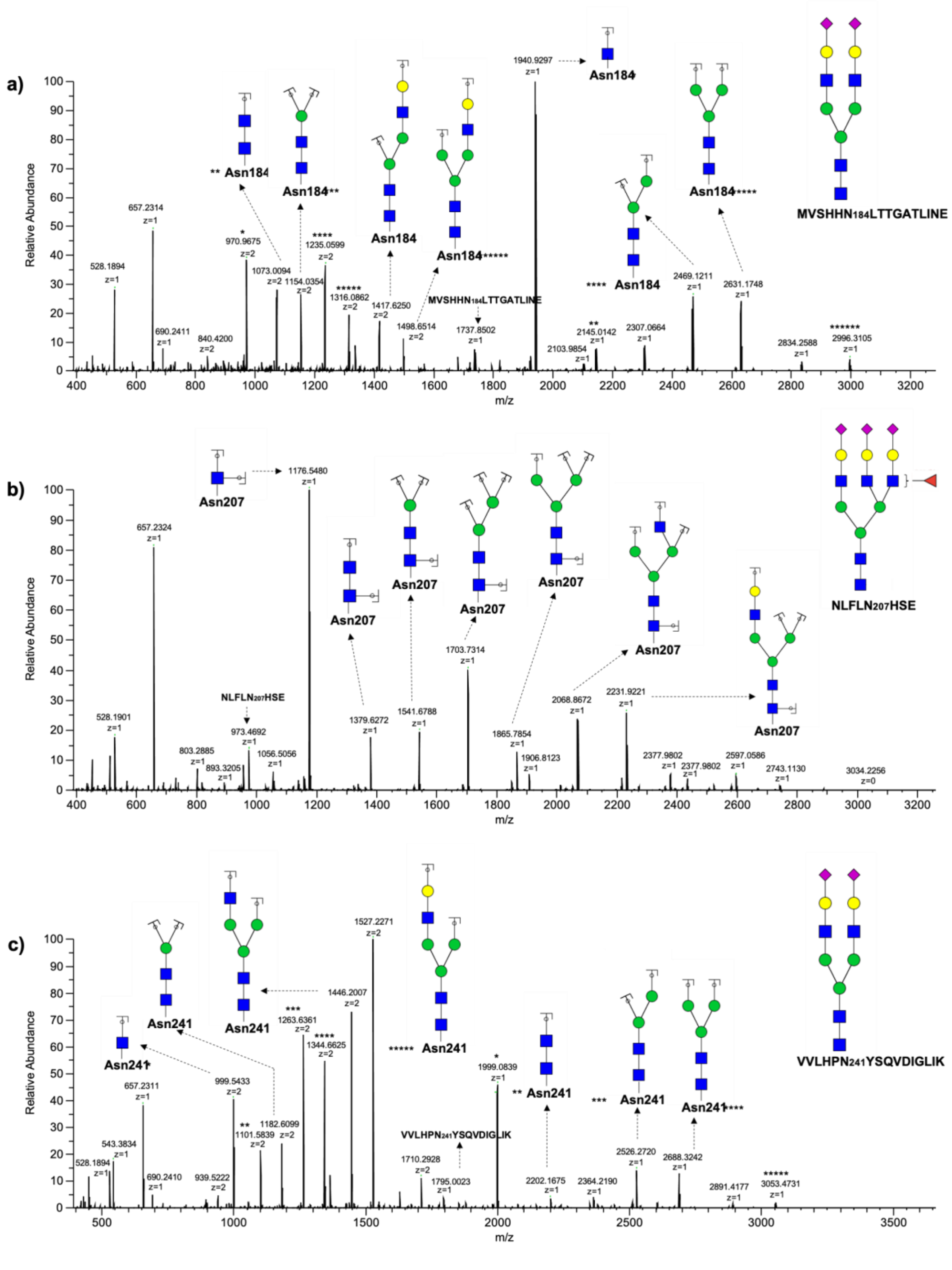

Supplementary Figure S1. Representative mass spectra of the three glycosylation sites. **a**) MVSHHN184LTTGATLINE = Asn184, **b**) NLFLN207HSE = Asn207, and **c**) VVLHPN241YSQVDIGLIK = Asn241. * Common fragment ion with different charge.

Supplementary Figure S2

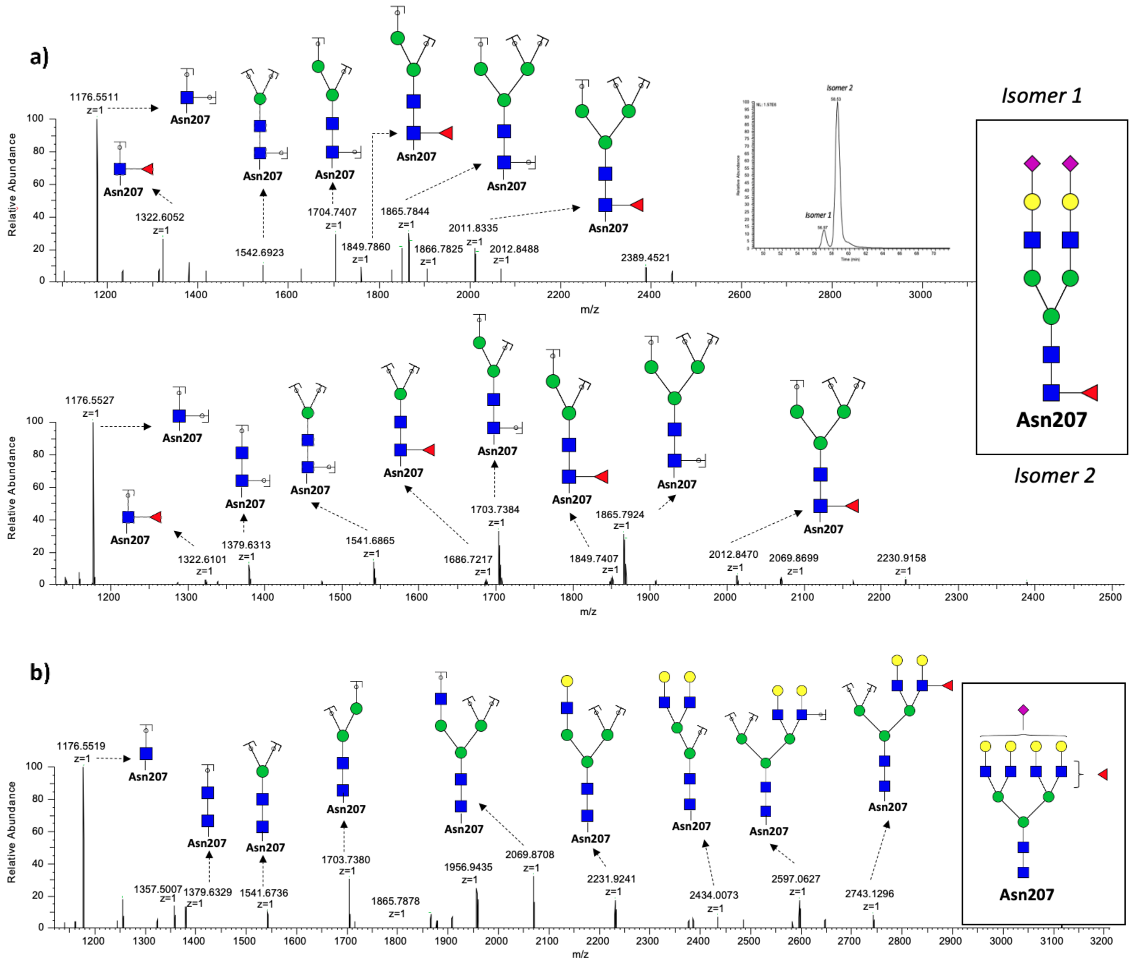

Supplementary Figure S2. Interpretation of fucosylated glycopeptides; **a**) *N*-glycopeptide NLFLN207HSE + 4-5-1-2 isomers 1 and 2, **b**) *N*-glycopeptide NLFLN207HSE + 6-7-1-1. NLFLN207HSE = Asn207.

The core or branch fucosylation of most abundant *N*-glycopeptides was performed by the evaluation of their fragmentation. The high abundance of core fragments allowed us to identified this type of structures, **Supplementary Figure S1a** shows the NLFLN207HSE + 4-5-1-2 **core** fucosylated glycopeptide where the core-fucose fragments were observed in both isomers. Otherwise, the low abundance of branched fragments made difficult the identification of this type of glycopeptides, **Supplementary Figure S1b** shows the NLFLN207HSE + 6-7-1-1 **branch** fucosylated glycopeptide, a branch-fucose fragment with a *m/z* of 2743.1296 was observed in the EIC, additionally the absence of core-fucose fragments gave us a clear conclusion of the branch fucosylation.

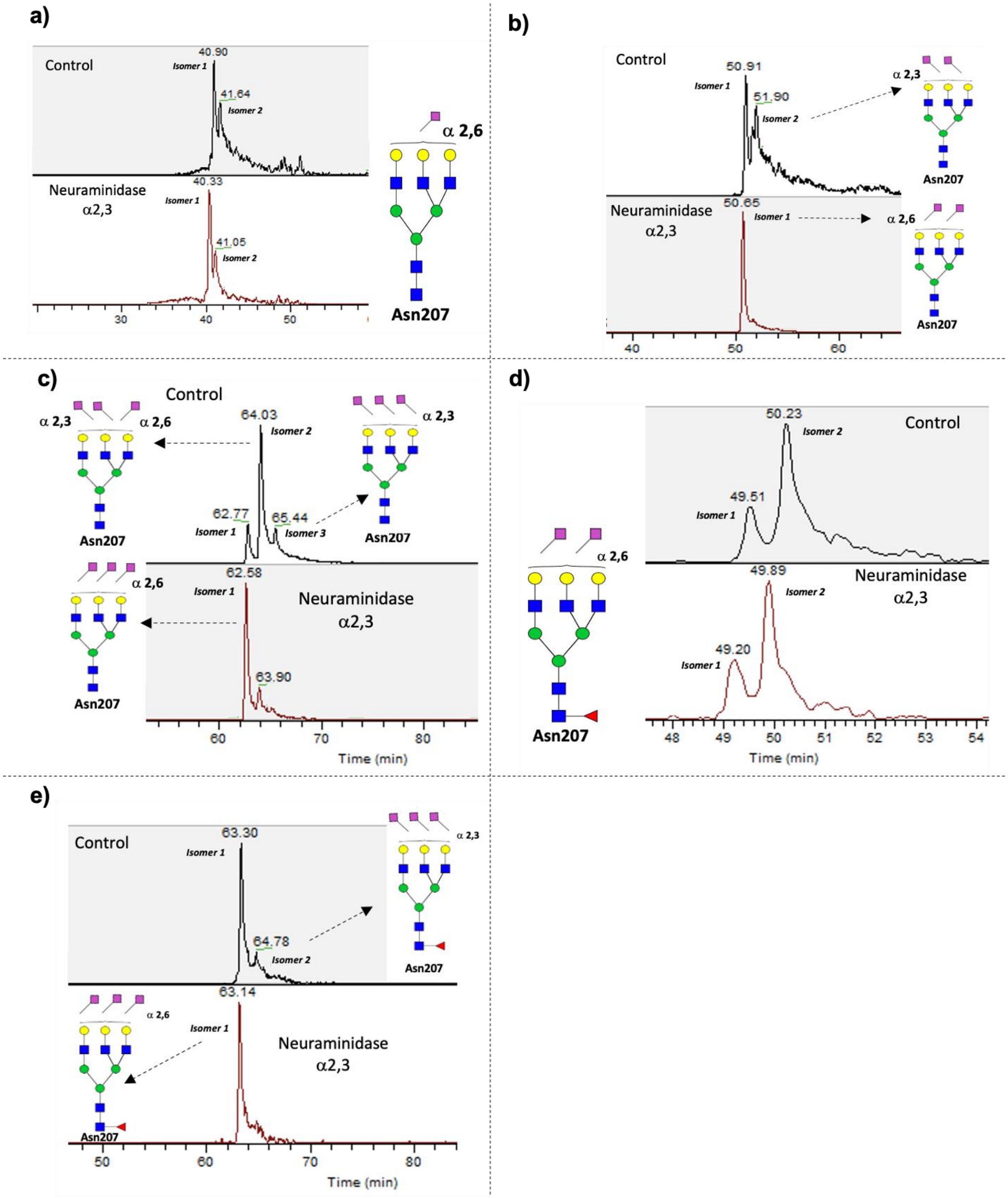

Supplementary Figure S3. Sialic acid linkage of important and abundant haptoglobin *N*-glycopeptides (NLFLN207HSE = Asn207).

A pooled sample was used to determine the sialic acid linkage of some abundant *N*-glycopeptides by the application of α2,3 neuraminidase enzyme digestion. The sample was fractionated using the LC method mentioned in section *2.4*, the fractions were collected considering the retention time ranges +/- 2 min aforementioned for the elution of mono-, di, and tri-sialylated glycopeptides (**Supplementary Table S1**). The fractionation of the sample was performed to avoid peak overlapping of the digested sialylated glycopeptide structures and the present sialylated glycopeptides in the sample, the fractions were divided in two, control and digested samples. The samples were analyzed in an scan range of 300 – 2000 *m/z*. We were able to identified the linkage of some high abundant sialylated glycopeptides. **a**) For the *N*-glycopeptide $NLFLN_{207}HSE$ + 5-6-0-1 both isomeric structures have sialic acid linkage α2,6, no changes were observed after enzymatic digestion. **b**) For the *N*-glycopeptide $NLFLN_{207}HSE$ + 5-6-0-2; the shift in mass of isomer 2 indicated an α2,3 linkages, and therefore no changes in the isomer 1 indicated α2,6 linkages. **c**) For the *N*-glycopeptide $NLFLN_{207}HSE$ + 5-6-0-3; isomer 1 have α2,6 linkages, the mass shift of isomer 3 after enzymatic digestion indicated a structure with α2,3 linkages. Meanwhile for isomer 2 the incomplete digestion indicated by a remaining peak suggest the presence of sialic acid molecules with both linkages, the change in peak area was about 1/3 of the initial which suggest a ratio 1 to 3 of linkages α2,6 and α2,3. Huang et al. reported these three isomeric structures in their glycomic analysis of serum haptoglobin [1]. **d**) For the *N*-glycopeptide $NLFLN_{207}HSE$ + 5612 both isomeric structures have sialic acid linkage α2,6, no changes were observed after enzymatic digestion. **e**) For the *N*-glycopeptide $NLFLN_{207}HSE$ + 5-6-1-3; the shift in mass of isomer 2 indicated an α2,3 linkages, and therefore no changes in the isomer 1 indicated α2,6 linkages.

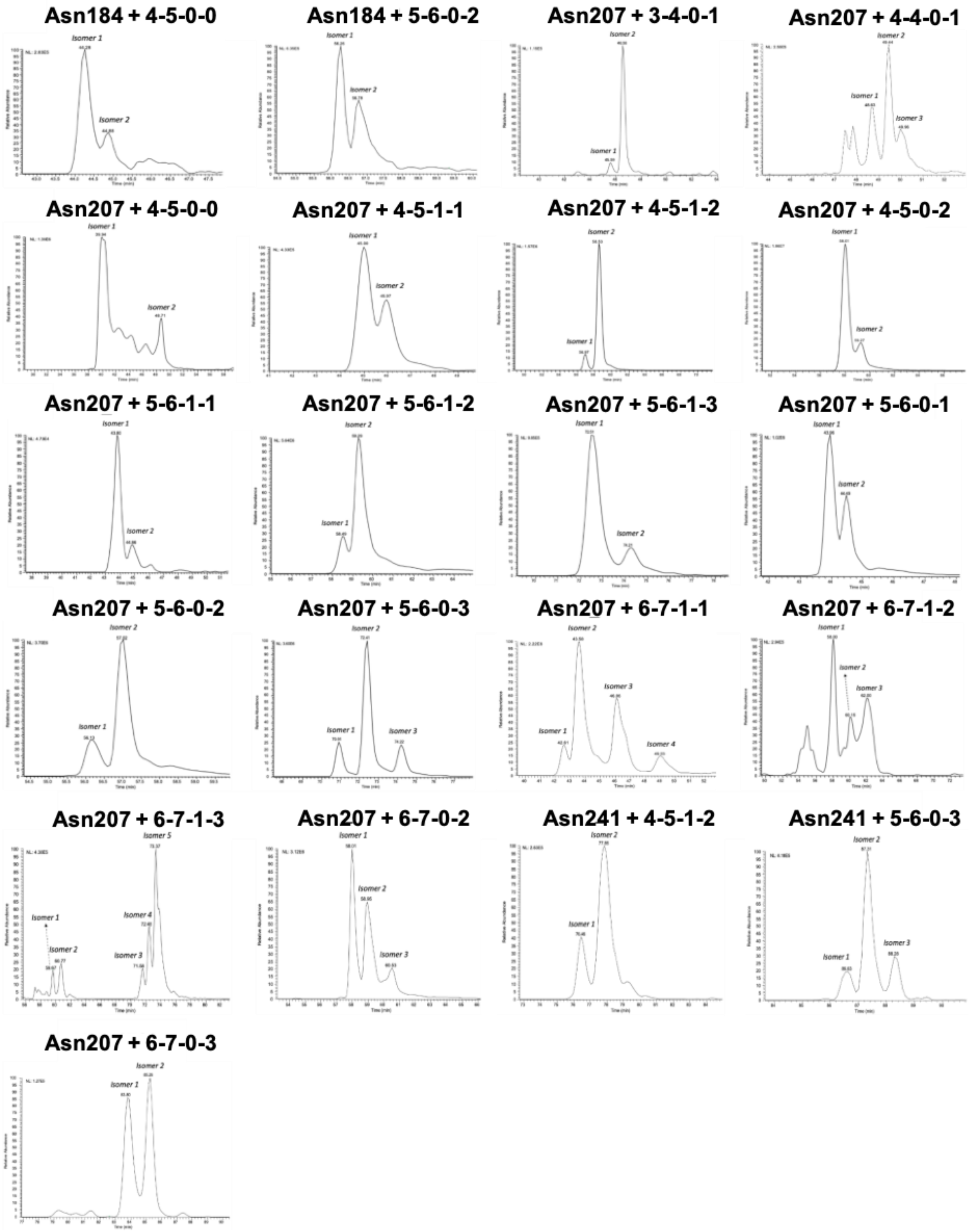

Supplementary Figure S4. EICs showing the achieved isomeric separation of important haptoglobin *N*-glycopeptides. MVSHHN$_{184}$LTTGATLINE = Asn184, NLFLN$_{207}$HSE = Asn207, and VVLHPN$_{241}$YSQVDIGLIK = Asn241.

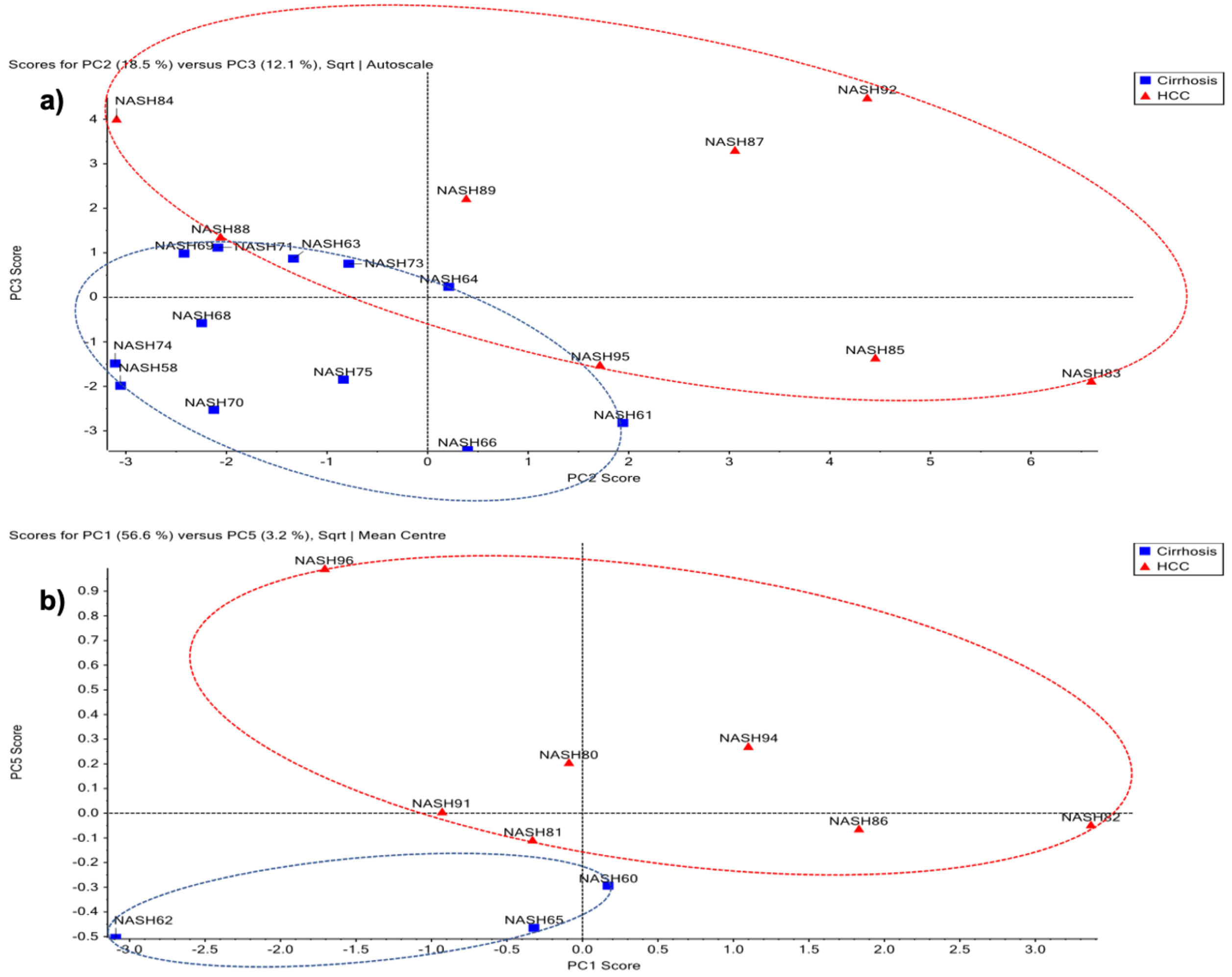

Supplementary Figure S5. PCA plot of *N*-glycopeptides derived from human serum haptoglobin from patients with cirrhosis and HCC, glycosylation site NLFLN207HSE. Gender evaluation, **a**) cirrhosis (female) “blue” vs. HCC (female) “red”, **b**) cirrhosis (male) “blue” vs. HCC (male) “red”.

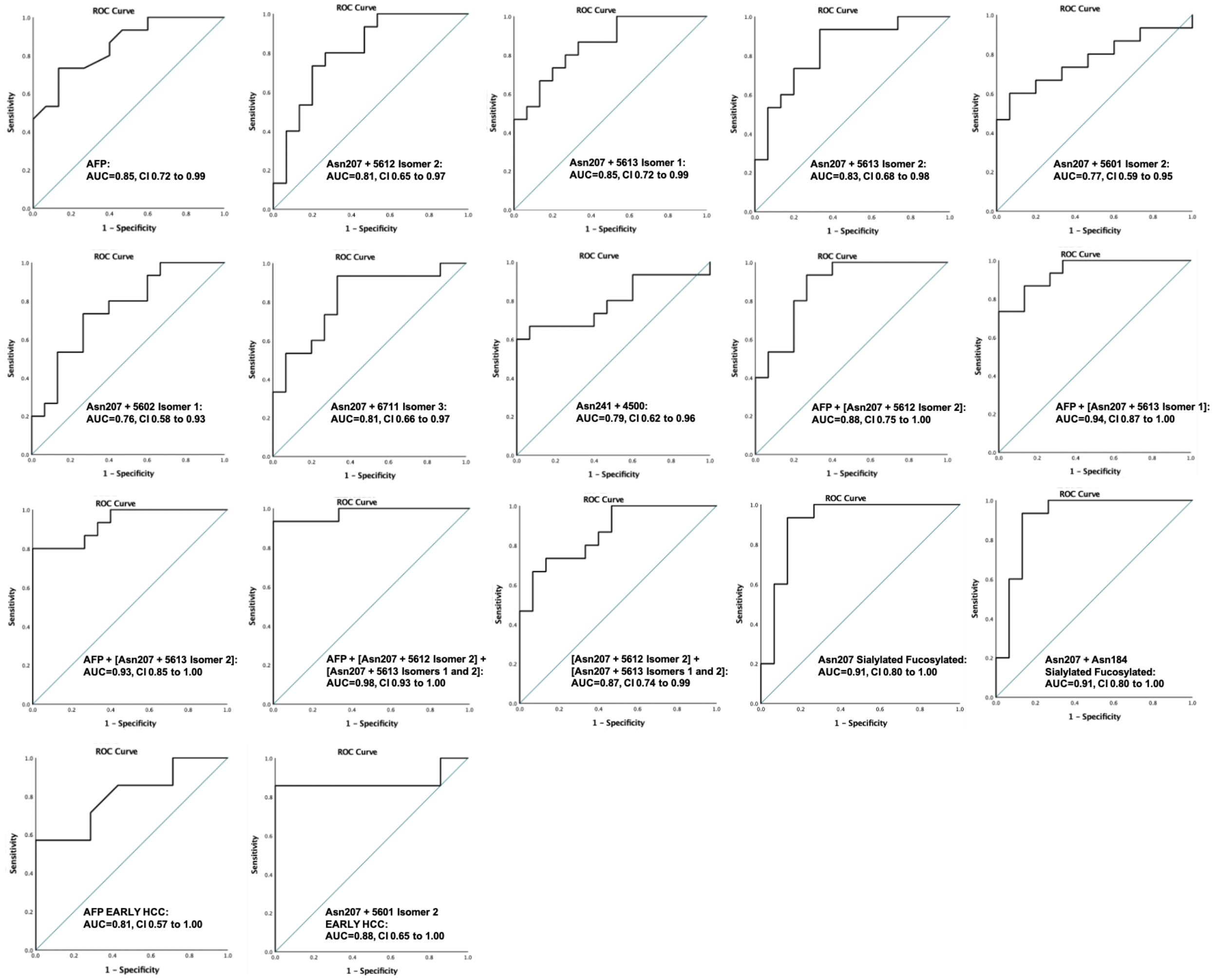

Supplementary Figure S6. Receiver operating characteristic (ROC) curve for the glycopeptide structures with statistically significance between cirrhosis and HCC patients. MVSHHN$_{184}$LTTGATLINE = Asn184, NLFLN$_{207}$HSE = Asn207, and VVLHPN$_{241}$YSQVDIGLIK = Asn241. Glycan nomenclature: HexNAc, Hex, Fuc, NeuAc (*N*-acetylhexosamine, Hexose, Fucose, *N*-acetylneuraminic acid).

Supplementary Table S1. PRM information for each of the target haptoglobin *N*-glycopeptides.

Glycopeptide [a, b]	Charge	rt (min)	Precursor ion (*m/z*)	Fragment ions (*m/z*)
Asn184 + 4-5-0-0	+ 3	42.24	1120.8183	1940.9333, 1072.5091, 1153.5359, 1234.5626, 1315.5888, 1417.1236
Asn184 + 4-5-1-1	+ 3	48.01	1266.5361	1940.9345, 1072.5099, 1153.5363, 1234.5613, 1315.5918, 1417.1268
Asn184 + 4-5-1-2	+ 3	57.07	1363.5679	1940.9336, 2086.9927, 2306.0684, 2468.1247, 2614.1797, 2630.1775
Asn184 + 4-5-0-1	+ 3	48.16	1217.8501	1940.9348, 1072.5088, 1153.5328, 1234.5603, 1315.5876, 1417.1272
Asn184 + 4-5-0-2	+ 3	54.73	1314.8819	1940.9337, 1072.5088, 1153.5358, 1234.5612, 1315.5887, 1498.1566
Asn184 + 4-6-0-1	+ 3	47.70	1271.8677	1940.9342, 2468.1247, 2630.1767, 2792.2461, 1336.5882, 1417.1265
Asn184 + 5-6-1-1	+ 3	47.18	1388.2468	1940.9331, 1072.5094, 1153.5355, 1234.5614, 1336.1014, 1417.1266
Asn184 + 5-6-1-2	+ 4	56.36	1114.2108	1940.9355, 2086.9979, 2306.0781, 2468.1318, 2630.1824, 2995.3198
Asn184 + 5-6-1-3	+ 3	57.30	1582.3104	1940.9361, 1459.6648, 1585.6160, 2101.8861, 2468.1117, 2995.3202
Asn184 + 5-6-0-1	+ 3	47.48	1339.5609	1940.9336, 1072.5086, 1153.5362, 1234.5614, 1315.5884, 1498.1562
Asn184 + 5-6-0-2	+ 3	54.08	1436.5927	1940.9337, 1072.5088, 1153.5349, 1234.5614, 1315.5827, 1417.1324
Asn207 + 3-4-0-1	+ 3	49.81	1533.6345	1176.5484, 1056.5070, 1379.6285, 1541.6803, 1703.7228, 1865.7870
Asn207 + 4-4-0-1	+ 2	49.44	1261.513	1176.5498, 1379.6307, 1541.6826, 1703.7349, 1865.7875, 1906.8068
Asn207 + 4-5-0-0	+ 2	40.39	1363.0527	1176.5490, 1379.6296, 1703.7336, 1865.7869, 2068.8884, 2230.9209
Asn207 + 4-5-1-1	+ 2	45.27	1298.5314	1176.5490, 1379.6298, 1541.6806, 1703.7309, 1865.7865, 2011.8485
Asn207 + 4-5-1-2	+ 3	57.98	1011.7411	1176.5505, 1703.7195, 1865.7773, 2068.8518, 2011.8485, 2230.9328
Asn207 + 4-5-0-1	+ 3	46.13	1108.7729	1176.5489, 1379.6279, 1541.6831, 1703.7328, 1865.7862, 2068.8710
Asn207 + 4-5-0-2	+ 2	57.49	1444.0791	1176.5495, 1541.6829, 1703.7334, 1865.7890, 2068.8653, 2230.9326
Asn207 + 5-6-1-1	+ 3	47.31	1060.0869	1176.5492, 1379.6293, 1541.6816, 1703.7332, 1865.7881, 2068.8665
Asn207 + 5-6-1-2	+ 3	56.34	1133.4518	1176.5496, 1379.6278, 1541.6826, 1703.7333, 1865.7822, 2068.8705
Asn207 + 5-6-1-3	+ 3	70.56	1230.4836	1176.5496, 1379.6244, 1541.6758, 1703.7313, 2068.8542, 2230.9204
Asn207 + 5-6-0-1	+ 3	45.18	1327.5154	1176.5497, 1379.6282, 1541.6810, 1703.7330, 1865.7879, 2230.9212
Asn207 + 5-6-0-2	+ 3	56.25	1084.7659	1176.5498, 1379.6283, 1541.6814, 1703.7339, 1865.7873, 2230.9204
Asn207 + 5-6-0-3	+ 3	71.39	1181.7977	1176.5502, 1379.6268, 1541.6714, 1703.7332, 2068.8577, 2230.9304
Asn207 + 6-7-1-1	+ 3	46.06	1278.8295	1176.5494, 1703.7345, 1932.9272, 2068.8702, 2230.9241, 1541.6814
Asn207 + 6-7-1-2	+ 3	58.07	1255.1626	1176.5503, 1129.5302, 1210.5564, 2068.8720, 2230.9256, 2596.0621
Asn207 + 6-7-1-3	+ 3	60.00	1352.1944	1176.5499, 1703.7356, 1875.9048, 1892.9331, 2068.8711, 2230.9238
Asn207 + 6-7-0-1	+ 3	43.81	1449.2262	1176.5494, 1379.6287, 1541.6718, 1703.7314, 2068.8716, 2596.0640
Asn207 + 6-7-0-2	+ 3	57.79	1206.4766	1176.5493, 1379.6289, 1703.7333, 2068.8687, 2230.9216, 2596.0574
Asn207 + 6-7-0-4	+ 3	60.29	1303.5084	1176.5509, 1201.5585, 1642.6395, 1516.6875, 1220.5885, 2528.0915
Asn241 + 4-4-0-1	+ 4	77.35	1123.4308	1998.0903, 2231.9111, 2069.8580, 1866.7745, 1177.5254, 1542.6679
Asn241 + 4-5-0-0	+ 3	61.74	1182.8833	1998.0914, 1101.0855, 1182.1120, 1263.1389, 1344.1667, 1445.7048
Asn241 + 4-5-1-2	+ 3	78.62	1139.8691	1998.0943, 2013.9092, 1290.7283, 1445.7031, 1486.7358, 1526.7318
Asn241 + 4-5-0-1	+ 3	67.98	1382.6187	1998.0868, 1101.0865, 1182.1102, 1344.1648, 1445.7029, 1526.7317
Asn241 + 4-5-0-2	+ 3	75.35	1236.9009	1999.0859, 1263.1281, 1344.1588, 1445.6924, 1526.7240, 1285.5670
Asn241 + 5-6-1-2	+ 3	78.15	1333.9327	1998.0907, 1128.6021, 1347.7465, 1445.6995, 1526.7316, 1709.3043
Asn241 + 5-6-0-1	+ 4	69.80	1128.4989	1998.0891, 1174.5344, 1417.6190, 1472.2158, 1580.6812, 1624.7572
Asn241 + 5-6-0-2	+ 4	77.58	1019.2106	1998.0902, 1263.1382, 1709.2971, 1344.1647, 1182.1115, 1628.2694
Asn241 + 5-6-0-3	+ 4	87.31	1091.9844	1998.0890, 1101.0842, 1263.1386, 1345.7034, 1526.7317, 1709.2975
Asn241 + 6-7-0-1	+ 4	69.23	1164.7583	1998.0898, 1209.6461, 1445.7010, 1827.7471, 1451.6415, 1288.5770
Asn241 + 6-7-0-2	+ 4	77.30	1110.4936	1998.0909, 1177.5336, 1445.7034, 1704.7222, 2231.9072, 2069.8579
Asn241 + 6-7-0-3	+ 4	87.23	1183.2675	1998.0868, 1645.7706, 1445.7043, 1255.5942, 1150.5383, 1037.4541

[a] **PEPTIDE_HexNAc, Hex, Fuc, NeuAc (*N*-acetylhexosamine, Hexose, Fucose, N-acetylneuraminic acid)**
[b] **MVSHHN$_{184}$LTTGATLINE = Asn184, NLFLN$_{207}$HSE = Asn207, and VVLHPN$_{241}$YSQVDIGLIK=Asn241**

Supplementary Table S2. Peak area (A) and normalized abundance (B) of all identified haptoglobin *N*-glycopeptides from patients with cirrhosis and hepatocellular carcinoma (HCC).

(A)

Glycosylation site MVSHHN$_{184}$LTTGATLINE

N - Glycopeptide	NASH58	NASH60	NASH61	NASH62	NASH63	NASH64	NASH65	NASH66	NASH68	NASH69	NASH70	NASH71	NASH73	NASH74	NASH75	NASH80	NASH81	NASH82	NASH83	NASH84	NASH85	NASH86	NASH87	NASH88	NASH89	NASH91	NASH92	NASH94	NASH95	NASH96
[Asn 184] + HexNAc4,Hex5 Isomer 1	7440	154319	4621175	1522050	3973189	2603015	524102	8292467	15050089	14828814	784821	2905063	3434550	32679	8797786	1698096	3959444	511973	5383989	27176	1686248	349657	6901076	490856	48479	137898	8049183	4616472	1089892	2932339
[Asn 184] + HexNAc4,Hex5 Isomer 2	0	28672	3092475	377597	1897375	387416	166082	3183069	5434431	5829589	269741	1589770	1407819	3810	4392390	493036	842703	55180	1520808	4818	683188	59709	986351	241135	10349	28960	5088920	3556940	539231	1034144
[Asn 184] + HexNAc4,Hex5,Fuc,Neu5Ac	1105699	740100	19253466	306400	1375615	2852840	864734	7242502	7317703	1361293	427685	2463004	5263267	89412	11839167	5922280	11341292	16436517	6526819	45552	12354964	1291930	9882707	39498	418556	775863	13863645	57079934	29252419	739278
[Asn 184] + HexNAc4,Hex5,Fuc,Neu5Ac2	186780	14922609	14135313	2244560	11077611	11099716	3992759	9809093	21635443	37755734	5229704	21617973	34126090	444510	26945436	49564919	38257658	69540935	23568314	854800	20153719	8667677	26495168	2804967	2507282	12195931	24805158	38040686	15079998	6601211
[Asn 184] + HexNAc4,Hex5,Neu5Ac	12778974	28620120	305965307	151752053	391334368	78595164	87180155	432364835	862983029	694364656	120058471	589268758	614727444	16573164	628692377	254760628	520217484	90849629	623532568	33666455	116905802	51923487	551728277	74445423	49487101	42704627	451503994	408126561	155201454	329515552
[Asn 184] + HexNAc4,Hex5,Neu5Ac2	74544907	735079986	836508554	341402197	995991689	596848831	309640288	901743819	1946322380	2811078145	501014814	1258228904	2208344173	65066310	1513981874	1810974665	2230007461	1077827358	1306303039	107014455	804857557	709676207	1799615592	404325485	225436961	426839734	1207220864	1135171183	575333795	1105548054
[Asn 184] + HexNAc4,Hex6,Neu5Ac	13179	551314	3074203	350232	1175134	983023	236576	3891616	6993869	10374093	286646	2807684	3384138	6376	6291877	3215537	3814552	1150162	1877381	33426	2908596	610008	3464046	60334	34097	180194	1930982	4861656	1055801	1260389
[Asn 184] + HexNAc5,Hex6,Fuc,Neu5Ac	79257	149258	3346718	746751	229138	120449	257509	862722	2497346	2997656	262489	1196697	466326	26216	3047275	1223422	1592533	2167433	943965	52413	3211047	133749	2628778	15150	99513	29239	1519604	7400311	2413099	145211
[Asn 184] + HexNAc5,Hex6,Fuc,Neu5Ac2	4728311	1338674	5949499	105133	385949	1870560	945469	1025464	3177214	3014438	276163	370010	667688	19683	4144450	3764970	6065259	9917909	13511839	32417	9305855	526342	5934786	31755	159913	287214	4648523	9212442	2520155	251249
[Asn 184] + HexNAc5,Hex6,Fuc,Neu5Ac3	801747	11221159	9473341	1877461	2455140	5221109	1618173	1537910	2869850	2769446	582181	2013034	8452160	58393	5004126	39217500	22770546	29361830	6557176	285536	2140878	6432428	26696072	750426	1567247	3012117	24264012	23493737	6229214	3137163
[Asn 184] + HexNAc5,Hex6,Neu5Ac	87502	1095730	16127029	3189034	5452995	3940848	1307080	20773159	21791372	22880571	1687750	7730686	11331014	120150	30244214	6099591	12244236	3251045	28777899	105225	8389910	709987	8455774	791975	780846	485743	13145626	17646249	3203244	3772312
[Asn 184] + HexNAc5,Hex6,Neu5Ac2 Isomer 1	756532	5843662	20937991	8273555	10581282	3847899	4650110	23661588	33739362	76494691	7939685	16853167	23854811	840574	53585231	22799252	45283200	2780024	38238571	943193	12443254	5587916	45569057	6871041	4139932	5350617	42164278	12578442	3423940	9055512
[Asn 184] + HexNAc5,Hex6,Neu5Ac2 Isomer 2	79887	3219467	12515748	6132631	9297283	7812783	1342506	12033485	23315074	20753183	2604468	22611721	15689804	250818	17630358	11952317	5817974	2901721	23296968	81831	9611615	503805	4320240	2107318	173097	985721	11381168	12520768	2632565	7283582
TOTAL	95170215	802965070	1255000819	518279654	1435226768	716183653	412725543	1426421729	2953127162	3704502309	641424618	1929656471	2931149284	83532095	2314596561	2211686213	2902214342	1306751716	2080039336	143147297	1004652633	786472902	2492677924	492975363	284863373	493013858	1809585957	1734305381	797974807	1471275996

Glycosylation site NLFLN$_{207}$HSE

N - Glycopeptide	NASH58	NASH60	NASH61	NASH62	NASH63	NASH64	NASH65	NASH66	NASH68	NASH69	NASH70	NASH71	NASH73	NASH74	NASH75	NASH80	NASH81	NASH82	NASH83	NASH84	NASH85	NASH86	NASH87	NASH88	NASH89	NASH91	NASH92	NASH94	NASH95	NASH96
[Asn 207] + HexNAc3,Hex4,Neu5Ac Isomer 1	16070	236663	3154005	700370	350715	1059870	431230	2498044	4980722	3619347	529286	1587355	1997557	14961	5749947	1778732	972118	1217835	338567137	191373	7326335	288028	1636615	333358	66496	241441	1760757	4630519	453826	1179324
[Asn 207] + HexNAc3,Hex4,Neu5Ac Isomer 2	496591	2342112	30553243	9493389	3965389	5665490	2638226	27552100	17857580	33054684	19402119	11057269	15087954	598434	58637628	13734319	25111050	3794664	60857723	1916704	30284216	3013131	57947356	3407840	1638112	9574787	10154473	18723542	12720416	8599894
[Asn 207] + HexNAc4,Hex4,Neu5Ac Isomer 1	41756	738379	1807440	2360430	442317	1300641	1039039	4399092	6601680	3736082	1570469	3854107	4971789	34428	8807697	2241167	2119101	465060	10195339	485243	4094196	659040	427397	411169	329141	2464069	2118185	4684426	923547	3009746
[Asn 207] + HexNAc4,Hex4,Neu5Ac Isomer 2	70942	937598	5673571	1676453	762183	1474884	859076	1918234	3098811	2687468	1273586	1694054	1876656	40909	5604083	1255913	399671	452533	1244452	422338	3315837	1146472	5359858	646756	288933	187387	3511397	2089245	420207	1274433
[Asn 207] + HexNAc4,Hex4,Neu5Ac Isomer 3	15253	505814	3795347	570130	219651	815548	342687	581672	939966	1892429	311849	759538	994316	7083	2426847	1179545	577241	483992	1966725	154675	3885923	470998	5254438	203966	189872	497912	1242529	2111608	235304	705821
[Asn 207] + HexNAc4,Hex5 Isomer 1	186780	5319287	78080775	26074996	16358120	28234523	6312509	50714573	106828893	72918615	7923188	46762096	60930304	205326	141259662	31725542	18876987	6494747	35022294	1391709	37749885	8841327	51172000	4243996	855483	5198368	34167957	47985521	11637498	44337120
[Asn 207] + HexNAc4,Hex5 Isomer 2	103872	1654326	19290772	8391616	4967490	8382814	2525799	16681857	31400544	15627055	3027662	7589363	11568547	221200	42753332	7022983	5867807	1699052	9147770	1022749	13977842	1743912	8453841	1287284	348393	1374834	6878740	14150649	2546132	11907233
[Asn 207] + HexNAc4,Hex5,Fuc, Neu5Ac Isomer 1	25933761	17929380	168597409	5525906	13011221	45461145	34864312	21299706	53327904	21916688	15966267	17004106	36688257	6015554	109799901	73844960	81535869	245785492	28708155	11342115	216127572	24240276	71732918	4041811	9696778	11935980	34551160	342099757	168022144	30211305
[Asn 207] + HexNAc4,Hex5,Fuc, Neu5Ac Isomer 2	4287384	9010181	33273873	2997280	12179150	19256055	11640733	11933010	46187133	17610340	7425144	9735907	13751772	1355872	31416500	12755594	24878539	27198863	14823924	12061843	40251947	14425423	32434277	3685845	6864671	5374848	13513858	98951973	18954627	14294163
[Asn 207] + HexNAc4,Hex5,Fuc, Neu5Ac2 Isomer 1	671045	8133920	16423985	4606983	8691573	11780645	3480536	16534392	18256150	45869376	6022713	19816915	28099607	698373	57169295	29606620	17460337	16182024	23325405	5250890	23616091	6322766	23799582	3125587	1812625	5289951	13684944	31237772	6797444	7327516
[Asn 207] + HexNAc4,Hex5,Fuc, Neu5Ac2 Isomer 2	4414552	38165185	58677009	4723372	26818359	76257114	19927810	14579522	82152795	58927501	7886466	19849729	43508491	2642407	76005415	56555824	61682627	132016780	67919932	36433708	141387356	625840080	85345125	8628976	23282823	19369658	53995907	204253901	45732188	39043008
[Asn 207] + HexNAc4,Hex5, Neu5Ac	23151508	241491565	2155865342	796313771	520580022	672743228	269112601	1484410472	2607455589	2669883496	319685864	1454662160	1694167014	31122664	3600862460	1118080068	795724500	260293175	1278374973	219542220	1206067587	368573627	1564784457	264016850	151363803	230796311	743614900	1482313094	429682433	1509549722
[Asn 207] + HexNAc4,Hex5, Neu5Ac2 Isomer 1	114541096	822334917	3457462975	896255918	913290878	2703992251	493415861	2127119643	4001859228	5333748878	738439137	1663119578	1942891970	98812309	5716125338	1955629521	1861118611	1002586986	1960685358	409145019	3963741190	818189232	1865122772	423442095	289158066	514113871	913218773	3526078788	675116410	1773901831
[Asn 207] + HexNAc4,Hex5, Neu5Ac2 Isomer 2	9356736	178125924	381298660	175433173	258081501	647324296	117909362	451749673	1023238063	1632970670	171514185	633748856	597314452	13123743	1250311745	307750797	372297230	100952691	419633475	148929087	470373549	216150629	590797190	105169690	81513337	167941822	334020483	445888766	89781617	375995095
[Asn 207] + HexNAc5,Hex6,Fuc, Neu5Ac Isomer 1	4474017	9755753	199689902	6232543	6921990	28989029	17552774	29111012	17754551	19212900	5788876	3009449	14384373	1940276	52761051	74204810	32906221	76524330	21134472	2507729	189781500	9038719	29519367	1407385	5774145	3268313	13003017	66467503	63446800	13086141
[Asn 207] + HexNAc5,Hex6,Fuc, Neu5Ac Isomer 2	341815	2700703	22218677	904997	1477993	13196088	3266875	5311206	8991690	5953452	1483034	2087779	4572086	167856	15023459	8710324	5103412	5445682	3621701	1026571	29604375	3170088	7311970	687252	923776	748815	3705549	7908800	6847643	3473571
[Asn 207] + HexNAc5,Hex6,Fuc, Neu5Ac2 Isomer 1	8850352	19034295	132152292	3564863	7292781	28949923	12816164	20346276	16928844	12670465	4648803	4466661	10515107	4200035	48320913	47186079	36037776	45114249	13411451	5333416	184003179	17113281	29529981	3611751	5804630	3641101	14945435	150545575	38382936	9157682
[Asn 207] + HexNAc5,Hex6,Fuc, Neu5Ac2 Isomer 2	14407674	89430849	300187455	32477849	78466671	172270037	80493506	68854894	155236124	119562991	24863373	85521926	125366092	8513614	260642214	271794324	216900141	213463921	149899805	56540297	556716890	111669436	286574464	20549419	45869334	45346225	190210479	614662538	101878353	132493513
[Asn 207] + HexNAc5,Hex6,Fuc, Neu5Ac3 Isomer 1	13664545	152927992	411737699	44650011	128896921	349599266	108183498	95891417	252957666	216932519	27021002	166445086	278952748	8847557	395867785	591499852	405142822	301621757	268007763	70829303	937487314	191739598	603811313	40891538	58990527	80101437	413119115	738020336	103849038	230611384
[Asn 207] + HexNAc5,Hex6,Fuc, Neu5Ac3 Isomer 2	1095784	20772855	47497269	5121182	19282778	82983825	12088953	15934594	57935562	47684366	3446985	27357128	42641131	727320	54454846	42060283	44914542	19092374	45530636	16725741	115568338	28911395	85268266	7425386	14046512	16659666	68279165	110011316	6650833	31604817
[Asn 207] + HexNAc5,Hex6, Neu5Ac Isomer 1	2048639	31554439	215016804	30899525	30761210	199836814	28940304	194061949	80843232	423590669	28030192	58838664	98977893	1768959	102630649	120282909	74573898	124697561	69693242	4188556	256981818	23081458	172110360	3926945	11950091	22047646	13382689	262404124	54751931	42720603
[Asn 207] + HexNAc5,Hex6, Neu5Ac Isomer 2	2863432	19035381	552061825	116139624	43633318	113127154	58228236	353170772	362851913	47509042	113585900	127465045	152611786	4976955	626453328	96126376	125356132	12191161	161318554	8708587	185564346	12824008	40635254	18613247	13450598	14245737	38638472	146354265	40436984	92604859
[Asn 207] + HexNAc5,Hex6, Neu5Ac2 Isomer 1	5357336	79245140	381272566	169151682	98266384	158495387	58774792	449985395	341897086	383298275	96540258	228227309	241460016	8038011	760898427	219056629	206961413	36007253	268377630	21890710	330213036	63960868	251643391	57645203	25552476	27181813	100773746	202222197	31509686	167224955
[Asn 207] + HexNAc5,Hex6, Neu5Ac2 Isomer 2	10144081	96606538	590132055	340623220	238932854	491496740	123281083	521045622	686168574	962320077	159276970	686957879	534886546	12264479	1563478717	280483408	303892803	55320289	657501470	61623886	339382921	90170818	392772609	117732727	69599685	108699399	180607040	358726869	42157364	368370962
[Asn 207] + HexNAc5,Hex6, Neu5Ac3 Isomer 1	3676278	65445184	233100262	86826942	63426750	152073028	24572731	200525850	192470917	310113733	33022965	161905976	201018071	3302483	498975250	221720726	166465724	43284480	221922466	16291989	394745180	68368636	300313313	39156010	13032616	26376640	124648284	187773033	26029496	123822294
[Asn 207] + HexNAc5,Hex6, Neu5Ac3 Isomer 2	2466268	101619363	472493885	315574576	265303931	553980744	82207601	487726824	642472666	1172815264	130967187	904353370	720937639	4867192	1652260657	293255974	298562481	4772026	751574106	36075086	300185260	85547324	305161483	181671320	48229953	115631351	182606717	183473541	10948967	412525373
[Asn 207] + HexNAc5,Hex6, Neu5Ac3 Isomer 3	641400	14510672	65007518	56120373	58645356	152238629	15405697	87998705	126228756	331339386	18816901	255242252	166769287	628393	337916599	39041894	65929080	2667707	229163180	13697865	55156518	13366323	96398171	30634665	16146066	32260243	32800234	36138751	2180263	86279175
[Asn 207] + HexNAc6,Hex7, Fuc, Neu5Ac Isomer 1	152131	949702	62668782	41226105	48041862	13220661	3274144	39185300	81268643	33523107	2291481	41315939	36978275	28676	17427847	14832111	10035839	4068834	92388886	4038294	25794602	2956694	31994984	3334654	3627765	1472472	19913006	88238470	41242196	50157141
[Asn 207] + HexNAc6,Hex7, Fuc, Neu5Ac Isomer 2	215334	4402697	11259525	11550654	15775334	15260783	4763317	26135702	25891677	15128414	1130967	8189742	13796150	628068	113535487	39744121	17794338	51120186	48837370	1609228	156065833	1772214	4991089	7558373	467024	580908	4990106	13485136	4358357	16807391
[Asn 207] + HexNAc6,Hex7, Fuc, Neu5Ac Isomer 3	218327	1334273	2462329	1128413	244825	1750158	2007889	1401790	2292075	2511877	2072597	3673484	4505068	455705	76840931	522227	312148	75363	677290	132095	3072890	1883591	1140313	173818	445838	142675	1273481	2014707	226932	527104
[Asn 207] + HexNAc6,Hex7, Fuc, Neu5Ac Isomer 4	788027	911452	2127067	1085726	454505	1469179	1319193	1584670	2623413	3738662	1522183	2148352	1694524	11620	22022558	1043833	265067	142810	1272428	280477	2762233	148949	2756397	276289	52562	694570	1717133	2308085	117812	1241425
[Asn 207] + HexNAc6,Hex7, Fuc, Neu5Ac2 Isomer 1	729817	8007092	39580307	1473799	4543007	27271950	7743226	9241583	4197997	8876713	1544137	2966886	8233920	238669	27247093	32501227	16353770	51447840	31415592	1244438	113075661	5894645	14422523	484871	3945949	2386451	11052731	81686780	14882268	4150912
[Asn 207] + HexNAc6,Hex7, Fuc, Neu5Ac2 Isomer 2	132736	1394055	9035437	1760934	1096695	3664510	980269	2825125	6112450	1932388	448375	1232293	2948528	479087	6894744	4709109	2474584	3227917	10836055	278791	6525815	1711908	5129569	465257	1127253	800176	5249262	11503917	1751056	1129250
[Asn 207] + HexNAc6,Hex7, Fuc, Neu5Ac2 Isomer 3	103126	1834119	2726387	3009645	883816	3146938	1343368	4352571	3972845	7179469	716771	3661676	3606892	110037	11620539	5272369	3997337	2238683	7701301	453420	6492377	1563641	8721346	954214	639980	1174648	4409658	7969999	2085701	2634601
[Asn 207] + HexNAc6,Hex7, Fuc, Neu5Ac3 Isomer 1	13341	164255	5820141	3492841	908595	1202208	102492	664896	431323	7427012	163007	1411822	1795821	7059	1428376	1366198	451197	389561	956408	20483	4224326	236898	1587601	97626	30720	221684	53335795	982557	30789	53013
[Asn 207] + HexNAc6,Hex7, Fuc, Neu5Ac3 Isomer 2	94604	271025	2005970	61131524	235917	310980	818458	2844766	3886733	1785409	1642086	1205587	1773876	6475	9460940	929632	1004928	725218	1931947	161437	1661261	336907	1794981	206126	95641	146352	833724	3064248	846133	1598518
[Asn 207] + HexNAc6,Hex7, Fuc, Neu5Ac3 Isomer 3	8651	596134	4755714	3464969	622357	722523	315298	848311	1375160	1333834	334822	1252417	1009679	26962	5314604	600778	386641	1674802	2067541	48208	2319778	167116	2278218	67286	51992	122007	1193751	4185892	169895	545557
[Asn 207] + HexNAc6,Hex7, Fuc, Neu5Ac3 Isomer 4	7545	462434	1528229	2475031	161518	1199060	1010595	1781233	3884559	3085752	958033	1673011	1511021	11811	7051491	1539252	972236	638857	2012422	491549	4069083	839419	5166320	499598	290667	624105	2219752	1899074	1152890	1469600
[Asn 207] + HexNAc6,Hex7, Fuc, Neu5Ac3 Isomer 5	79498	2300727	7604837	8803381	1872436	11224323	1967005	1902127	2183966	3789304	323498	2158404	4925238	59413	8865034	7528196	3683501	5588032	20144150	455325	24708016	2403041	6867696	394778	2134255	2371143	9176194	15284730	1609131	1592620
[Asn 207] + HexNAc6,Hex7, Neu5Ac	1067730	20792628	217872851	60870877	22613778	72762122	16415007	163949295	44111760	77993733	18107306	38747124	68238867	1578891	208766248	103545977	50387459	18280089	347958682	2098143	212578272	10101574	63619263	6145540	8536294	8991010	61017263	129127688	24136148	28526924
[Asn 207] + HexNAc6,Hex7, Neu5Ac2 Isomer 1	182205	5569409	36615396	8327851	4515863	24935892	4457998	35474092	9422949	15916805	3620340	7926731	13949150	517782	44332734	24047151	13462502	9629261	91582594	878888	76149504	3013887	14844086	875752	2237928	2632551	8478057	32633854	4611047	5188107
[Asn 207] + HexNAc6,Hex7, Neu5Ac2 Isomer 2	202454	11667467	63642191	22361496	15740026	95437020	11099316	67648015	23409406	34054122	9030631	35087609	37918228	486950	48753322	35762965	27759967	4158108	222190957	1959111	75123843	5306690	15159462	4323262	6441600	14521535	13863213	47039874	4613461	14202024
[Asn 207] + HexNAc6,Hex7, Neu5Ac2 Isomer 3	356257	4883843	20199788	6286535	5040238	21455483	3399030	16160584	22895885	91855410	2306348	13786720	14244279	90220	58730991	21177388	10428872	1056216	110307201	1023708	24829660	5089766	24677438	1961907	1709665	4186280	10399668	15810749	1060403	6755915
[Asn 207] + HexNAc6,Hex7, Neu5Ac4	204972	188682	4617726	3365448	602725	2042080	4786150	3036788	4139905	5322037	520855	1396455	959574	7217	4680897	1818627	289851	2986476	7874361	144816	4550029	398738	2718260	446841	279791	306625	1851347	3062057	675626	1079494
TOTAL	257875722	2095294409	10531045292	3385226403	2944381003	7018061038	1656114750	7140973354	11189023385	14282919816	1995203818	6770953809	7265030551	219881035	18149587581	6215526334	5411428370	2897274937	8111784323	1179047820	10781593384	2852692571	7173187314	1374864263	928893866	1511944817	3754128146	9750206226	2095663932	5672971136

Glycosylation site VVLHPN$_{241}$YSQVDIGLIK

N - Glycopeptide	NASH58	NASH60	NASH61	NASH62	NASH63	NASH64	NASH65	NASH66	NASH68	NASH69	NASH70	NASH71	NASH73	NASH74	NASH75	NASH80	NASH81	NASH82	NASH83	NASH84	NASH85	NASH86	NASH87	NASH88	NASH89	NASH91	NASH92	NASH94	NASH95	NASH96
[Asn 241] + HexNAc4,Hex4,Neu5Ac	112332	988165	7895375	1931208	1718938	4271568	1858149	5053490	3825256	9554470	338863	1484328	2145853	778891	10658988	1569840	1042499	522371	8391075	314774	4129567	294605	2114839	642212	200860	422475	1481112	2834260	492023	1528320
[Asn 241] + HexNAc4,Hex5	0	36878	28950	135148	736212	109919	69370	399022	149671	230328	131217	130029	250017	18126	219872	96897	60156	82439	114403	9056	79933	18204	79010	7240	45481	17106	11098	24619	154837	280125
[Asn 241] + HexNAc4,Hex5,Fuc, Neu5Ac2 Isomer 1	322913	41180	4611479	2139897	3517046	1727540	1594082	1544769	2041832	3911714	329899	1483229	1498806	210866	4919838	3149185	1553867	1956140	3760090	1213452	5435646	2278928	3542011	3185465	132536	593708	29365227	2885243	4434620	1458174
[Asn 241] + HexNAc4,Hex5,Fuc, Neu5Ac2 Isomer 2	1873036	5103180	6270522	7020115	10547282	5923979	7494638	5222921	13565296	19581868	3305607	16464649	14917385	866878	17510148	13982341	9836729	25323907	23053221	4993321	9244466	7762124	12757548	4603102	2720021	4994492	25109259	7069686	9175651	14382499
[Asn 241] + HexNAc4,Hex5, Neu5Ac	14366917	23670741	11424619	28824016	152889125	13199138	50575814	22198091	25888804	42671002	35917267	31131532	51924930	15693897	28335061	44130895	56686390	40058778	36702685	25473659	15878972	43316866	51922097	41120739	9799806	24404158	40277107	15306213	42229964	85917837
[Asn 241] + HexNAc4,Hex5, Neu5Ac2	263738795	412164944	144852788	453456859	2247346236	119604664	615950349	199601118	376617159	456919619	386899716	423224292	826818644	290753435	310166380	669038371	933882790	747077130	641996078	604911584	193483233	690701708	745142557	755594020	188668379	442690891	621110692	140433588	660309197	1415608846
[Asn 241] + HexNAc5,Hex6,Fuc, Neu5Ac2	3604282	9000128	25003466	28187200	21218237	17815264	7572827	19084198	29765386	22380558	7631149	16243866	17071578	603911	50154578	17784583	1167438	39397818	44223029	15442921	48322475	10676374	3458514	2634827	4509914	4608401	47195953	22029828	23945565	28105148
[Asn 241] + HexNAc5,Hex6, Neu5Ac	681126	4430368	10456298	9017220	4688519	393684	3614915	39384950	21165097	10120711	2066098	46845291	26972507	1638808	25608863	13777398	7960032	10133827	9114080	613780	18828914	7734381	10989151	8014348	7828054	980762	18815739	5768495	27990252	12729483
[Asn 241] + HexNAc5,Hex6, Neu5Ac2	1074422	7819760	5773061	9653433	36157433	6173894	25923583	7898353	7357762	7455186	10125147	2973105	6307533	1138064	5942275	7053490	13179252	22328958	19899177	3162317	7848195	5642756	10730373	36622487	1584262	4698854	17207182	4346483	7242132	7319069
[Asn 241] + HexNAc5,Hex6, Neu5Ac3 Isomer 1	772788	3650114	1900300	2512438	10984513	1823621	6445224	1861228	2174052	2298688	2580630	819486	1316034	599133	2450813	2813508	6286389	8586268	9331454	2794117	3031056	4329029	4130019	6483241	820150	1960870	4977171	1431422	1588175	1818370
[Asn 241] + HexNAc5,Hex6, Neu5Ac3 Isomer 2	907761	10436414	2623733	18440384	75136310	5972595	26743783	5765566	8354955	8289779	8984921	13183995	16686115	1213400	9852149	12720526	27398808	30367711	40832523	8866204	5532636	10839692	13443404	27887628	2501273	17680391	23423146	2384535	4864572	18542775
[Asn 241] + HexNAc5,Hex6, Neu5Ac3 Isomer 3	129834	1885319	1064587	2975683	17925884	3983289	7424039	1696338	2204190	3757065	2654876	3450435	4293483	107802	3253008	3027427	5255825	5505844	18957722	1712114	1718898	1468378	2175368	1477633	350456	9372957	5914110	1053250	1269682	4324841
[Asn 241] + HexNAc6,Hex7, Neu5Ac	79749	437216	4243806	2527691	1409921	1408794	1271545	2780895	4353030	3977473	949538	1836160	1794709	112138	4406748	1536992	1276292	539383	3454983	319444	3155826	373580	2903488	743386	633133	479321	3197262	3269513	1543644	1668037
[Asn 241] + HexNAc6,Hex7, Neu5Ac2	82132	648241	6536674	3111290	2202997	4824167	3339595	7136495	6249313	8947082	1126014	2246024	3445913	182924	17538019	3801264	1723399	729040	15827144	315041	6896683	503596	3285420	1005165	174898	751175	2979271	3923887	944688	2409989
[Asn 241] + HexNAc6,Hex7, Neu5Ac3 Isomer 1	736902	3505652	14596718	3097003	5555297	1368524	949822	2845845	4475118	3046714	1608050	5682974	7739990	598515	4042827	3510333	3658598	1856156	17655715	2147950	2535511	2516822	5375302	5119285	4445886	3362673	6063033	1519672	2664634	5000228
[Asn 241] + HexNAc6,Hex7, Neu5Ac3 Isomer 2	796223	3540139	2261707	4611062	5397347	8006198	4145213	6963349	13088824	25945327	2278564	16689328	8570486	635330	14008264	8685777	5353405	2292115	27940028	5535231	16628701	5749622	22177104	5062976	4908607	4054060	6403365	12006889	4885737	7151744
TOTAL	289279212	487358439	249544083	577640647	2597431297	196606838	764972948	329436628	521275745	629087584	466927556	583888723	991753983	315152118	509067831	806678827	1076321869	936757885	921253407	677824965	342750712	794206665	894226205	900203754	229323716	521072294	853530727	226287583	793735373	1608245485

(B)

Glycosylation site MVSHHN$_{184}$LTTGATLINE

N - Glycopeptide	NASH58	NASH60	NASH61	NASH62	NASH63	NASH64	NASH65	NASH66	NASH68	NASH69	NASH70	NASH71	NASH73	NASH74	NASH75	NASH80	NASH81	NASH82	NASH83	NASH84	NASH85	NASH86	NASH87	NASH88	NASH89	NASH91	NASH92	NASH94	NASH95	NASH96
[Asn 184] + HexNAc4,Hex5 Isomer 1	0.007818	0.019219	0.368221	0.293674	0.276834	0.363456	0.126986	0.581347	0.509632	0.400292	0.122356	0.150548	0.117174	0.039121	0.380100	0.076778	0.136428	0.039179	0.258841	0.018985	0.167844	0.044459	0.276854	0.099570	0.017018	0.027970	0.444808	0.266186	0.136582	0.199306
[Asn 184] + HexNAc4,Hex5 Isomer 2	0.000000	0.003571	0.246412	0.072856	0.132200	0.054095	0.040240	0.223151	0.184023	0.157365	0.042053	0.082386	0.048030	0.004561	0.189769	0.022292	0.029037	0.004223	0.073114	0.003366	0.068002	0.007592	0.039570	0.048914	0.003633	0.005874	0.281220	0.205093	0.067575	0.070289
[Asn 184] + HexNAc4,Hex5,Fuc,Neu5Ac	1.161812	0.092171	1.534140	0.059119	0.095847	0.398339	0.209518	0.507739	0.247795	0.036747	0.066677	0.127640	0.179563	0.107039	0.511500	0.267772	0.390781	1.257815	0.313783	0.031822	1.229775	0.164269	0.396469	0.008012	0.146932	0.157371	0.766122	3.291227	3.665832	0.050247
[Asn 184] + HexNAc4,Hex5,Fuc,Neu5Ac2	0.196259	1.858438	1.126319	0.433079	0.771837	1.549842	0.967413	0.687671	0.732628	1.019185	0.815326	1.120302	1.164256	0.532143	1.164153	2.241047	1.318223	5.321664	1.133071	0.597147	2.006039	1.102095	1.062920	0.568987	0.880170	2.473750	1.370764	2.193425	1.889784	0.448673
[Asn 184] + HexNAc4,Hex5,Neu5Ac	13.427493	3.564304	24.379690	29.279956	27.266379	10.974163	21.123034	30.311150	29.222684	18.743804	18.717472	30.537496	20.972233	19.840474	27.162072	11.518841	17.924847	6.952325	29.976960	23.518750	11.636440	6.602069	22.133958	15.101246	17.372223	8.661953	24.950680	23.532566	19.449418	22.396583
[Asn 184] + HexNAc4,Hex5,Neu5Ac2	78.327980	91.545699	66.654025	65.872197	69.396120	83.337399	75.023292	63.217196	65.907165	75.882748	78.109695	65.204814	75.340556	77.893784	65.410184	81.882080	76.838138	82.481419	62.801843	74.758279	80.113019	90.235303	72.196074	82.017382	79.138627	86.577634	66.712546	65.453939	72.099243	75.142125
[Asn 184] + HexNAc4,Hex6,Neu5Ac	0.013848	0.068660	0.244956	0.067576	0.081878	0.137259	0.057320	0.272824	0.236829	0.280040	0.044689	0.145502	0.115454	0.007633	0.271835	0.145388	0.131436	0.088017	0.090257	0.023351	0.289513	0.077562	0.138969	0.012239	0.011970	0.036549	0.106708	0.280323	0.132310	0.085666
[Asn 184] + HexNAc5,Hex6,Fuc,Neu5Ac	0.083279	0.018588	0.266671	0.144083	0.015965	0.016818	0.062392	0.060482	0.084566	0.080919	0.040923	0.062016	0.015909	0.031384	0.131655	0.055316	0.054873	0.165864	0.045382	0.036615	0.319618	0.017006	0.105460	0.003073	0.034934	0.005931	0.083975	0.426702	0.302403	0.009870
[Asn 184] + HexNAc5,Hex6,Fuc,Neu5Ac2	4.968268	0.166716	0.474063	0.020285	0.026891	0.261184	0.229079	0.071891	0.107588	0.081372	0.043055	0.019175	0.022779	0.023563	0.179057	0.170231	0.208987	0.758974	0.649595	0.022646	0.926276	0.066924	0.238089	0.006441	0.056137	0.058257	0.256883	0.531189	0.315819	0.017077
[Asn 184] + HexNAc5,Hex6,Fuc,Neu5Ac3	0.842435	1.397465	0.754847	0.362249	0.171063	0.729018	0.392070	0.107816	0.097180	0.074759	0.090764	0.104321	0.288357	0.069905	0.216199	1.773195	0.784592	2.246933	0.315243	0.199470	0.213096	0.817883	1.070980	0.152224	0.550175	0.610960	1.340860	1.354648	0.780628	0.213227
[Asn 184] + HexNAc5,Hex6,Neu5Ac	0.091943	0.136460	1.285021	0.615311	0.379940	0.550257	0.316695	1.456313	0.737908	0.617642	0.263125	0.400625	0.386572	0.143837	1.306673	0.275789	0.421893	0.248788	1.383527	0.073508	0.835106	0.090275	0.339224	0.160652	0.274112	0.098525	0.726444	1.017482	0.401422	0.256397
[Asn 184] + HexNAc5,Hex6,Neu5Ac2 Isomer 1	0.794925	0.727760	1.668365	1.596350	0.737255	0.537278	1.126683	1.658807	1.142496	2.064911	1.237820	0.873377	0.813838	1.006289	2.315100	1.030854	1.560298	0.212743	1.838358	0.658897	1.238563	0.710503	1.828117	1.393790	1.453304	1.085287	2.330051	0.725273	0.429079	0.615487
[Asn 184] + HexNAc5,Hex6,Neu5Ac2 Isomer 2	0.083941	0.400947	0.997270	1.183267	0.647792	1.090891	0.325278	0.843613	0.789505	0.560215	0.406044	1.171800	0.535278	0.300265	0.761703	0.540416	0.200467	0.222056	1.120025	0.057166	0.956710	0.064059	0.173317	0.427469	0.060765	0.199938	0.628938	0.721947	0.329906	0.495052
TOTAL	100	100	100	100	100	100	100	100	100	100	100	100	100	100	100	100	100	100	100	100	100	100	100	100	100	100	100	100	100	100

Glycosylation site NLFLN$_{207}$HSE

N - Glycopeptide	NASH58	NASH60	NASH61	NASH62	NASH63	NASH64	NASH65	NASH66	NASH68	NASH69	NASH70	NASH71	NASH73	NASH74	NASH75	NASH80	NASH81	NASH82	NASH83	NASH84	NASH85	NASH86	NASH87	NASH88	NASH89	NASH91	NASH92	NASH94	NASH95	NASH96
[Asn 207] + HexNAc3,Hex4,Neu5Ac Isomer 1	0.006232	0.011295	0.029950	0.020689	0.011911	0.015102	0.026039	0.034982	0.044514	0.025340	0.026528	0.023444	0.027496	0.006804	0.031681	0.028618	0.017964	0.042034	4.173769	0.016231	0.067952	0.010097	0.022816	0.024247	0.007159	0.015969	0.046902	0.047491	0.021655	0.020788
[Asn 207] + HexNAc3,Hex4,Neu5Ac Isomer 2	0.192570	0.111780	0.290125	0.280436	0.134676	0.080727	0.159302	0.385831	0.159599	0.231428	0.972438	0.163304	0.207679	0.272163	0.323080	0.220968	0.464037	0.130974	0.750238	0.162564	0.280888	0.105624	0.807833	0.247867	0.176351	0.633276	0.270488	0.192032	0.606987	0.151594
[Asn 207] + HexNAc4,Hex4,Neu5Ac Isomer 1	0.016192	0.035240	0.017163	0.069727	0.015022	0.018533	0.062740	0.061604	0.059001	0.026158	0.078712	0.056921	0.068435	0.015658	0.048528	0.036058	0.039160	0.016052	0.125686	0.041155	0.037974	0.023102	0.005958	0.029906	0.035434	0.162973	0.056423	0.048044	0.044069	0.053054
[Asn 207] + HexNAc4,Hex4,Neu5Ac Isomer 2	0.027510	0.044748	0.053875	0.049523	0.025886	0.021016	0.051873	0.026862	0.027695	0.018816	0.063832	0.025019	0.025831	0.018605	0.030877	0.020206	0.007386	0.015619	0.015341	0.035820	0.030755	0.040189	0.074721	0.047041	0.031105	0.012394	0.093534	0.021428	0.020051	0.022465
[Asn 207] + HexNAc4,Hex4,Neu5Ac Isomer 3	0.005915	0.024140	0.036040	0.016842	0.007460	0.011621	0.020692	0.008146	0.008401	0.013250	0.015630	0.011218	0.013686	0.003221	0.013371	0.018977	0.010667	0.016705	0.024245	0.013119	0.036042	0.016511	0.073251	0.014835	0.020441	0.032932	0.033098	0.021657	0.011228	0.012442
[Asn 207] + HexNAc4,Hex5 Isomer 1	0.072430	0.253868	0.741434	0.770259	0.555571	0.402312	0.381164	0.710191	0.954765	0.510530	0.397112	0.690628	0.838679	0.093380	0.778308	0.510424	0.348836	0.224167	0.431746	0.118037	0.350133	0.309929	0.713379	0.308685	0.092097	0.343820	0.910144	0.492149	0.555313	0.781550
[Asn 207] + HexNAc4,Hex5 Isomer 2	0.040280	0.078954	0.183180	0.247889	0.168711	0.119446	0.152514	0.233608	0.280637	0.109411	0.151747	0.112087	0.159236	0.100600	0.235561	0.112991	0.108434	0.058643	0.112771	0.086744	0.129645	0.061132	0.117853	0.093630	0.037506	0.090931	0.183231	0.145132	0.121495	0.209894
[Asn 207] + HexNAc4,Hex5,Fuc, Neu5Ac Isomer 1	10.056690	0.855697	1.600956	0.163236	0.441900	0.647774	2.105187	0.298275	0.476609	0.153447	0.800232	0.251133	0.504998	2.735822	0.604972	1.188073	1.506735	8.483333	0.353907	0.961972	2.004598	0.849733	1.000015	0.293979	1.043906	0.789445	0.920351	3.508641	8.017609	0.532548
[Asn 207] + HexNAc4,Hex5,Fuc, Neu5Ac Isomer 2	1.662578	0.430020	0.315960	0.088540	0.413640	0.274379	0.702894	0.167106	0.412790	0.123296	0.372150	0.143789	0.189287	0.616639	0.173098	0.205221	0.459741	0.938774	0.182746	1.023016	0.373340	0.505677	0.452160	0.268088	0.739016	0.355492	0.359973	1.014871	0.904469	0.251970
[Asn 207] + HexNAc4,Hex5,Fuc, Neu5Ac2 Isomer 1	0.260220	0.388199	0.155958	0.136091	0.295192	0.167862	0.210163	0.231543	0.163161	0.321148	0.301860	0.292675	0.386779	0.317614	0.314989	0.476333	0.322657	0.558526	0.287550	0.445350	0.219041	0.221642	0.331785	0.227338	0.195138	0.349877	0.364531	0.320381	0.324358	0.129165
[Asn 207] + HexNAc4,Hex5,Fuc, Neu5Ac2 Isomer 2	1.711891	1.821471	0.557181	0.139529	0.910832	1.086584	1.203287	0.204167	0.734227	0.412573	0.395271	0.293160	0.598876	1.201744	0.418772	0.909912	1.139859	4.556584	0.837300	3.090096	1.311377	21.938574	1.189780	0.627624	2.506511	1.281109	1.438308	2.094867	2.182229	0.688229
[Asn 207] + HexNAc4,Hex5, Neu5Ac	8.977777	11.525424	20.471523	23.523206	17.680457	9.585885	16.249635	20.787229	23.303692	18.692841	16.022717	21.483859	23.319475	14.154319	19.839913	17.988502	14.704519	8.984069	15.759479	18.620298	11.186358	12.920201	21.814354	19.203121	16.295059	15.264863	19.807925	15.202890	20.503404	26.609508
[Asn 207] + HexNAc4,Hex5, Neu5Ac2 Isomer 1	44.417169	39.246748	32.831147	26.475509	31.018094	38.529050	29.793579	29.787531	35.765939	37.343547	37.010712	24.562560	26.743067	44.938987	31.494519	31.463619	34.392373	34.604482	24.170827	34.701308	36.763965	28.681297	26.001311	30.798829	31.129290	34.003481	24.325722	36.164146	32.214918	31.269361
[Asn 207] + HexNAc4,Hex5, Neu5Ac2 Isomer 2	3.628390	8.501236	3.620710	5.182317	8.765221	9.223691	7.119637	6.326164	9.145017	11.433031	8.596324	9.359817	8.221775	5.968565	6.888926	4.951323	6.879833	3.484401	5.173134	12.631302	4.362746	7.577074	8.236188	7.649460	8.775312	11.107669	8.897418	4.573121	4.284161	6.627834
[Asn 207] + HexNAc5,Hex6,Fuc, Neu5Ac Isomer 1	1.734951	0.465603	1.896202	0.184110	0.235092	0.413063	1.059877	0.407662	0.158678	0.134517	0.290140	0.044446	0.197995	0.882421	0.290701	1.193862	0.608088	2.641252	0.260540	0.212691	1.760236	0.316849	0.411524	0.102365	0.621615	0.216166	0.346366	0.681704	3.027527	0.230675
[Asn 207] + HexNAc5,Hex6,Fuc, Neu5Ac Isomer 2	0.132550	0.128894	0.210983	0.026734	0.050197	0.188030	0.197261	0.074376	0.080362	0.041682	0.074330	0.030834	0.062933	0.076339	0.082776	0.140138	0.094308	0.187959	0.044647	0.087068	0.274583	0.111126	0.101935	0.049987	0.099449	0.049527	0.098706	0.081114	0.326753	0.061230
[Asn 207] + HexNAc5,Hex6,Fuc, Neu5Ac2 Isomer 1	3.432022	0.908431	1.254883	0.105306	0.247685	0.412506	0.773869	0.284923	0.151299	0.088711	0.232999	0.065968	0.144736	1.910140	0.266237	0.759165	0.665957	1.557127	0.165333	0.452349	1.706642	0.599899	0.411672	0.262699	0.624897	0.240822	0.398107	1.544025	1.831541	0.161427
[Asn 207] + HexNAc5,Hex6,Fuc, Neu5Ac2 Isomer 2	5.587061	4.268176	2.850500	0.959400	2.664963	2.454667	4.860382	0.964223	1.387397	0.837105	1.246157	1.263071	1.725610	3.871918	1.436078	4.372829	4.008186	7.367748	1.847926	4.795420	5.163586	3.914528	3.995078	1.494651	4.938060	2.999198	5.066702	6.304098	4.861388	2.335522
[Asn 207] + HexNAc5,Hex6,Fuc, Neu5Ac3 Isomer 1	5.298888	7.298640	3.909751	1.318967	4.377726	4.981422	6.532367	1.342834	2.260766	1.518825	1.354298	2.458222	3.839664	4.023793	2.181139	9.516489	7.486800	10.410533	3.303931	6.007331	8.695258	6.721355	8.417615	2.974224	6.350621	5.297907	11.004396	7.569279	4.955424	4.065090
[Asn 207] + HexNAc5,Hex6,Fuc, Neu5Ac3 Isomer 2	0.424927	0.991405	0.451021	0.151280	0.654901	1.182432	0.729959	0.223143	0.517789	0.333856	0.172764	0.404037	0.586937	0.330779	0.300034	0.676697	0.829994	0.658977	0.561290	1.418580	1.071904	1.013477	1.188708	0.540081	1.512176	1.101870	1.818776	1.128297	0.317362	0.557112
[Asn 207] + HexNAc5,Hex6, Neu5Ac Isomer 1	0.794429	1.505967	2.041742	0.912776	1.044743	2.847465	1.747482	2.717584	0.722523	2.965715	1.404879	0.868986	1.362388	0.804507	0.565471	1.935201	1.378082	4.303960	0.859160	0.355249	2.383524	0.809111	2.399357	0.285624	1.286486	1.458231	0.356479	2.691267	2.612629	0.753055
[Asn 207] + HexNAc5,Hex6, Neu5Ac Isomer 2	1.110392	0.908482	5.242232	3.430779	1.481918	1.611943	3.515954	4.945695	3.242927	0.332628	5.692947	1.882527	2.100635	2.263476	3.451612	1.546552	2.316507	0.420780	1.988694	0.738612	1.721122	0.449540	0.566488	1.353824	1.448023	0.942213	1.029226	1.501038	1.929555	1.632387
[Asn 207] + HexNAc5,Hex6, Neu5Ac2 Isomer 1	2.077488	3.782053	3.620463	4.996761	3.337421	2.258393	3.548956	6.301457	3.055647	2.683613	4.838616	3.370682	3.323593	3.655618	4.192373	3.524346	3.824525	1.242797	3.308491	1.856643	3.062748	2.242123	3.508111	4.192792	2.750850	1.797805	2.684345	2.074030	1.503566	2.947749
[Asn 207] + HexNAc5,Hex6, Neu5Ac2 Isomer 2	3.933709	4.610643	5.603737	10.062051	8.114876	7.003312	7.443994	7.296563	6.132515	6.737558	7.982992	10.145659	7.362482	5.577779	8.614404	4.512625	5.615760	1.909390	8.105510	5.226581	3.147799	3.160902	5.475566	8.563225	7.492749	7.189376	4.810892	3.679172	2.011647	6.493440
[Asn 207] + HexNAc5,Hex6, Neu5Ac3 Isomer 1	1.425601	3.123436	2.213458	2.564878	2.154162	2.166881	1.483758	2.808102	1.720176	2.171221	1.655117	2.391184	2.766927	1.501941	2.749237	3.567208	3.076188	1.493972	2.735803	1.381792	3.661288	2.396635	4.186609	2.847991	1.403025	1.744550	3.320299	1.925837	1.242064	2.182671
[Asn 207] + HexNAc5,Hex6, Neu5Ac3 Isomer 2	0.956379	4.849885	4.486676	9.322111	9.010516	7.893644	4.963883	6.829977	5.741991	8.211313	6.564101	13.356366	9.923394	2.213557	9.103571	4.718120	5.517258	0.164707	9.265213	3.059680	2.784238	2.998827	4.254196	13.213764	5.192192	7.647855	4.864158	1.881740	0.522458	7.271769
[Asn 207] + HexNAc5,Hex6, Neu5Ac3 Isomer 3	0.248724	0.692536	0.617294	1.657803	1.991772	2.169241	0.930231	1.232307	1.128148	2.319829	0.943107	3.769665	2.295507	0.285788	1.861842	0.628135	1.218330	0.092076	2.825065	1.161773	0.511580	0.468551	1.343868	2.228196	1.738204	2.133692	0.873711	0.370646	0.104037	1.520882
[Asn 207] + HexNAc6,Hex7, Fuc, Neu5Ac Isomer 1	0.058994	0.045325	0.595086	1.217824	1.631646	0.188381	0.197700	0.548739	0.726325	0.234708	0.114849	0.610194	0.508990	0.013042	0.096023	0.238630	0.185456	0.140437	1.138947	0.342505	0.239247	0.103646	0.446036	0.242544	0.390547	0.097389	0.530430	0.904991	1.967978	0.884142
[Asn 207] + HexNAc6,Hex7, Fuc, Neu5Ac Isomer 2	0.083503	0.210123	0.106917	0.341208	0.535778	0.217450	0.287620	0.365996	0.231402	0.105920	0.056684	0.120954	0.189898	0.285640	0.625554	0.639433	0.328829	1.764423	0.602055	0.136485	1.447521	0.062124	0.069580	0.549754	0.050277	0.038421	0.132923	0.138306	0.207970	0.296271
[Asn 207] + HexNAc6,Hex7, Fuc, Neu5Ac Isomer 3	0.084664	0.063679	0.023382	0.033333	0.008315	0.024938	0.121241	0.019630	0.020485	0.017587	0.103879	0.054254	0.062010	0.207251	0.423376	0.008402	0.005768	0.002601	0.008349	0.011204	0.028501	0.066029	0.015897	0.012643	0.047997	0.009437	0.033922	0.020663	0.010829	0.009291
[Asn 207] + HexNAc6,Hex7, Fuc, Neu5Ac Isomer 4	0.305584	0.043500	0.020198	0.032072	0.015436	0.020934	0.079656	0.022191	0.023446	0.026176	0.076292	0.031729	0.023324	0.005285	0.121339	0.016794	0.004898	0.004929	0.015686	0.023788	0.025620	0.005221	0.038426	0.020096	0.005659	0.045939	0.045740	0.023672	0.005622	0.021883
[Asn 207] + HexNAc6,Hex7, Fuc, Neu5Ac2 Isomer 1	0.283011	0.382146	0.375844	0.043536	0.154294	0.388597	0.467554	0.129416	0.037519	0.062149	0.077392	0.043818	0.113336	0.108545	0.150125	0.522904	0.302208	1.775732	0.387283	0.105546	1.048784	0.206634	0.201062	0.035267	0.424801	0.157840	0.294415	0.837795	0.710146	0.073170
[Asn 207] + HexNAc6,Hex7, Fuc, Neu5Ac2 Isomer 2	0.051473	0.066533	0.085798	0.052018	0.037247	0.052215	0.059191	0.039562	0.054629	0.013529	0.022473	0.018200	0.040585	0.217885	0.037988	0.075764	0.045729	0.111412	0.133584	0.023645	0.060527	0.060010	0.071510	0.033840	0.121354	0.052924	0.139826	0.117986	0.083556	0.019906
[Asn 207] + HexNAc6,Hex7, Fuc, Neu5Ac2 Isomer 3	0.039991	0.087535	0.025889	0.088905	0.030017	0.044841	0.081116	0.060952	0.035507	0.050266	0.035925	0.054079	0.049647	0.050044	0.064026	0.084826	0.073868	0.077269	0.094940	0.038456	0.060217	0.054813	0.121583	0.069404	0.068897	0.077691	0.117462	0.081742	0.099525	0.046441
[Asn 207] + HexNAc6,Hex7, Fuc, Neu5Ac3 Isomer 1	0.005173	0.007839	0.055267	0.103179	0.030859	0.017130	0.006189	0.009311	0.003855	0.051999	0.008170	0.020851	0.024719	0.003210	0.007870	0.021980	0.008338	0.013446	0.011790	0.001737	0.039181	0.008304	0.022132	0.007101	0.003307	0.014662	1.420724	0.010077	0.001469	0.000934
[Asn 207] + HexNAc6,Hex7, Fuc, Neu5Ac3 Isomer 2	0.036686	0.012935	0.019048	1.805833	0.008012	0.004431	0.049420	0.039837	0.034737	0.012500	0.082302	0.017805	0.024417	0.002945	0.052128	0.014957	0.018570	0.025031	0.023817	0.013692	0.015408	0.011810	0.025023	0.014992	0.010296	0.009680	0.022208	0.031428	0.040375	0.028178
[Asn 207] + HexNAc6,Hex7, Fuc, Neu5Ac3 Isomer 3	0.003355	0.028451	0.045159	0.102356	0.021137	0.010295	0.019038	0.011879	0.012290	0.009339	0.016781	0.018497	0.013898	0.012262	0.029282	0.009666	0.007145	0.057806	0.025488	0.004089	0.021516	0.005858	0.031760	0.004894	0.005597	0.008070	0.031798	0.042931	0.008107	0.009617
[Asn 207] + HexNAc6,Hex7, Fuc, Neu5Ac3 Isomer 4	0.002926	0.022070	0.014512	0.073113	0.005486	0.017085	0.061022	0.024944	0.034718	0.021604	0.048017	0.024709	0.020799	0.005372	0.038852	0.024765	0.017966	0.022050	0.024809	0.041690	0.037741	0.029425	0.072023	0.036338	0.031292	0.041278	0.059128	0.019477	0.055013	0.025905
[Asn 207] + HexNAc6,Hex7, Fuc, Neu5Ac3 Isomer 5	0.030828	0.109804	0.072214	0.260053	0.063594	0.159935	0.118772	0.026637	0.019519	0.026530	0.016214	0.031877	0.067794	0.027021	0.048844	0.121119	0.068069	0.192872	0.248332	0.038618	0.229169	0.084238	0.095741	0.028714	0.229763	0.156827	0.244429	0.156763	0.076784	0.028074
[Asn 207] + HexNAc6,Hex7, Neu5Ac	0.414048	0.992349	2.068863	1.798133	0.768032	1.036784	0.991176	2.295896	0.394241	0.546063	0.907542	0.572255	0.939278	0.718066	1.150253	1.665925	0.931130	0.630941	4.289546	0.177952	1.971678	0.354107	0.886904	0.446992	0.918974	0.594665	1.625338	1.324359	1.151718	0.502857
[Asn 207] + HexNAc6,Hex7, Neu5Ac2 Isomer 1	0.070656	0.265806	0.347690	0.246006	0.153372	0.355310	0.269184	0.496768	0.084216	0.111439	0.181452	0.117070	0.192004	0.235483	0.244263	0.386888	0.248779	0.332356	1.129007	0.074542	0.706292	0.105651	0.206938	0.063697	0.240924	0.174117	0.225833	0.334699	0.220028	0.091453
[Asn 207] + HexNAc6,Hex7, Neu5Ac2 Isomer 2	0.078508	0.556841	0.604329	0.660561	0.534578	1.359877	0.670202	0.947322	0.209218	0.238425	0.452617	0.518208	0.521928	0.221461	0.268619	0.575381	0.512988	0.143518	2.739113	0.166160	0.696779	0.186024	0.211335	0.314450	0.693470	0.960454	0.369279	0.482450	0.220143	0.250345
[Asn 207] + HexNAc6,Hex7, Neu5Ac2 Isomer 3	0.138151	0.233086	0.191812	0.185705	0.171182	0.305718	0.205241	0.226308	0.204628	0.643114	0.115595	0.203616	0.196066	0.041031	0.323594	0.340718	0.192719	0.036455	1.359839	0.086825	0.230297	0.178420	0.344023	0.142698	0.184054	0.276880	0.277020	0.162158	0.050600	0.119090
[Asn 207] + HexNAc6,Hex7, Neu5Ac4	0.079485	0.009005	0.043849	0.099416	0.020470	0.029097	0.288999	0.042526	0.037000	0.037262	0.026105	0.020624	0.013208	0.003282	0.025791	0.029259	0.005356	0.103079	0.097073	0.012282	0.042202	0.013978	0.037895	0.032501	0.030121	0.020280	0.049315	0.031405	0.032239	0.019029
TOTAL	100	100	100	100	100	100	100	100	100	100	100	100	100	100	100	100	100	100	100	100	100	100	100	100	100	100	100	100	100	100

Glycosylation site VVLHPN$_{241}$YSQVDIGLIK

N - Glycopeptide	NASH58	NASH60	NASH61	NASH62	NASH63	NASH64	NASH65	NASH66	NASH68	NASH69	NASH70	NASH71	NASH73	NASH74	NASH75	NASH80	NASH81	NASH82	NASH83	NASH84	NASH85	NASH86	NASH87	NASH88	NASH89	NASH91	NASH92	NASH94	NASH95	NASH96
[Asn 241] + HexNAc4,Hex4,Neu5Ac	0.038832	0.202759	3.163920	0.334327	0.066178	2.172645	0.242904	1.533979	0.733826	1.518782	0.072573	0.254214	0.216369	0.247148	2.093825	0.194605	0.096858	0.055764	0.910832	0.046439	1.204831	0.037094	0.236499	0.071341	0.087588	0.081078	0.173528	1.252504	0.061988	0.095030
[Asn 241] + HexNAc4,Hex5	0.000000	0.007567	0.011601	0.023397	0.028344	0.055908	0.009068	0.121123	0.028712	0.036613	0.028102	0.022269	0.025210	0.005752	0.043191	0.012012	0.005589	0.008800	0.012418	0.001336	0.023321	0.002292	0.008836	0.000804	0.019833	0.003283	0.001300	0.010880	0.019507	0.017418
[Asn 241] + HexNAc4,Hex5,Fuc, Neu5Ac2 Isomer 1	0.111627	0.008450	1.847962	0.370455	0.135405	0.878677	0.208384	0.468912	0.391699	0.621808	0.070653	0.254026	0.151127	0.066909	0.966441	0.390389	0.144368	0.208820	0.408149	0.179021	1.585889	0.286944	0.396098	0.353860	0.057794	0.113940	3.440442	1.275034	0.558703	0.090669
[Asn 241] + HexNAc4,Hex5,Fuc, Neu5Ac2 Isomer 2	0.647484	1.047110	2.512791	1.215308	0.406066	3.013109	0.979726	1.585410	2.602326	3.112741	0.707949	2.819827	1.504142	0.275067	3.439649	1.733322	0.913921	2.703357	2.502376	0.736668	2.697140	0.977343	1.426658	0.511340	1.186105	0.958503	2.941811	3.124204	1.156009	0.894297
[Asn 241] + HexNAc4,Hex5, Neu5Ac	4.966453	4.856947	4.578197	4.989956	5.886166	6.713468	6.611451	6.738198	4.966432	6.782999	7.692257	5.331758	5.235666	4.979785	5.566068	5.470690	5.266676	4.276321	3.983994	3.758147	4.632805	5.454105	5.806372	4.567937	4.273350	4.683450	4.718882	6.764053	5.320408	5.342333
[Asn 241] + HexNAc4,Hex5, Neu5Ac2	91.171015	84.571213	58.046974	78.501550	86.521874	60.834438	80.519233	60.588623	72.249124	72.632115	82.860759	72.483724	83.369329	92.258125	60.928301	82.937391	86.766126	79.751358	69.687241	89.243037	56.450133	86.967503	83.328195	83.935889	82.271639	84.957672	72.769576	62.059785	83.190093	88.021938
[Asn 241] + HexNAc5,Hex6,Fuc, Neu5Ac2	1.245953	1.846716	10.019659	4.879712	0.816893	9.061365	0.989947	5.792980	5.710104	3.557622	1.634333	2.782014	1.721352	0.191625	9.852239	2.204667	0.108466	4.205763	4.800311	2.278305	14.098432	1.344282	0.386761	0.292692	1.966615	0.884407	5.529497	9.735323	3.016820	1.747566
[Asn 241] + HexNAc5,Hex6, Neu5Ac	0.235456	0.909057	4.190161	1.561043	0.180506	0.200239	0.472555	11.955243	4.060250	1.608792	0.442488	8.022983	2.719677	0.520005	5.030540	1.707916	0.739559	1.081798	0.989313	0.090551	5.493472	0.973850	1.228901	0.890282	3.413539	0.188220	2.204459	2.549188	3.526396	0.791514
[Asn 241] + HexNAc5,Hex6, Neu5Ac2	0.371413	1.604519	2.313443	1.671183	1.392046	3.140223	3.388823	2.397533	1.411491	1.185079	2.168462	0.509190	0.635998	0.361116	1.167286	0.874386	1.224471	2.383642	2.160011	0.466539	2.289768	0.710490	1.199962	4.068244	0.690841	0.901766	2.016000	1.920778	0.912411	0.455097
[Asn 241] + HexNAc5,Hex6, Neu5Ac3 Isomer 1	0.267143	0.748959	0.761509	0.434948	0.422899	0.927547	0.842543	0.564973	0.417064	0.365400	0.552683	0.140350	0.132698	0.190109	0.481432	0.348777	0.584062	0.916594	1.012908	0.412218	0.884333	0.545076	0.461854	0.720197	0.357639	0.376314	0.583127	0.632568	0.200089	0.113065
[Asn 241] + HexNAc5,Hex6, Neu5Ac3 Isomer 2	0.313801	2.141425	1.051411	3.192363	2.892716	3.037837	3.496043	1.750129	1.602790	1.317746	1.924264	2.257964	1.682485	0.385020	1.935331	1.576901	2.545596	3.241789	4.432279	1.308037	1.614187	1.364845	1.503356	3.097924	1.090717	3.393078	2.744265	1.053763	0.612871	1.152982
[Asn 241] + HexNAc5,Hex6, Neu5Ac3 Isomer 3	0.044882	0.386844	0.426613	0.515144	0.690139	2.026018	0.970497	0.514921	0.422845	0.597224	0.568584	0.590941	0.432918	0.034206	0.639013	0.375295	0.488314	0.587755	2.057818	0.252589	0.501501	0.184886	0.243268	0.164144	0.152822	1.798782	0.692899	0.465448	0.159963	0.268917
[Asn 241] + HexNAc6,Hex7, Neu5Ac	0.027568	0.089711	1.700624	0.437589	0.054281	0.716554	0.166221	0.844137	0.835072	0.632261	0.203359	0.314471	0.180963	0.035582	0.865650	0.190533	0.118579	0.057580	0.375031	0.047128	0.920735	0.047038	0.324693	0.082580	0.276087	0.091987	0.374592	1.444849	0.194478	0.103718
[Asn 241] + HexNAc6,Hex7, Neu5Ac2	0.028392	0.133011	2.619447	0.538620	0.084814	2.453713	0.436564	2.166272	1.198850	1.422232	0.241154	0.384666	0.347456	0.058043	3.445124	0.471224	0.160119	0.077826	1.718001	0.046478	2.012157	0.063409	0.367404	0.111660	0.076267	0.144159	0.349053	1.734027	0.119018	0.149852
[Asn 241] + HexNAc6,Hex7, Neu5Ac3 Isomer 1	0.254737	0.719317	5.849354	0.536147	0.213877	0.696071	0.124164	0.863852	0.858493	0.484307	0.344390	0.973297	0.780434	0.189913	0.794163	0.435159	0.339917	0.198147	1.916488	0.316889	0.739754	0.316898	0.601112	0.568681	1.938694	0.645337	0.710347	0.671567	0.335708	0.310912
[Asn 241] + HexNAc6,Hex7, Neu5Ac3 Isomer 2	0.275244	0.726393	0.906336	0.798258	0.207796	4.072187	0.541877	2.113714	2.510921	4.124279	0.487991	2.858306	0.864175	0.201595	2.751748	1.076733	0.497380	0.244686	3.032828	0.816617	4.851544	0.723945	2.480033	0.562426	2.140471	0.778023	0.750221	5.306031	0.615537	0.444692
TOTAL	100	100	100	100	100	100	100	100	100	100	100	100	100	100	100	100	100	100	100	100	100	100	100	100	100	100	100	100	100	100

Supplementary Table S3. Complete clinical information.

NASH labels	Cirrhosis/HCC	AFP	MELD score	TNM	Gender	Race	Age	Etiology	Lab Date	Sodium	Creatinine	Albumin	Total biliribun	INR	Ascites: 1-none 2-Mild 3-Severe	Encephalopathy: 1-none 2-Cont 3-Uncont	Child Pugh (CTP) Score	No. Lesions	Max diameter (cm)	Portal vein invasion	Lymph node involvement	Evidence of metastases	Presence of cirrhosis	ALT	AST	ALKPHOS	NA	WBC	PLT	ALBI score	Infiltrative	Hepatic vein thrombosis	Location of mets	Smoking	Alcohol use	Diabetes	Functional status (ECOG)	Cancer history	Family history of HCC
**58**	Cirrhosis	5.7	8	*No data*	Female	Caucasian	67.0	NASH	1/8/2018	142	1.21	4.1	0.6	1.0	1	1	5	*No data*	*No data*	*No data*	*No data*	*No data*	*No data*	31	46	155	*No data*	*No data*	147	*No data*	*No data*	*No data*	*No data*	*No data*	*No data*	*No data*	*No data*	*No data*	*No data*
**60**	Cirrhosis	*No data*	9	*No data*	Male	Caucasian	64.0	NASH	1/11/2018	136	1.25	2.9	1.0	1.0	1	1	6	*No data*	*No data*	*No data*	*No data*	*No data*	*No data*	33	38	129	*No data*	*No data*	189	*No data*	*No data*	*No data*	*No data*	*No data*	*No data*	*No data*	*No data*	*No data*	*No data*
**61**	Cirrhosis	3.0	8	*No data*	Female	Hispanic	52.0	NASH	1/18/2018	134	0.92	3.9	0.3	1.2	1	1	5	*No data*	*No data*	*No data*	*No data*	*No data*	*No data*	55	54	236	*No data*	*No data*	85	*No data*	*No data*	*No data*	*No data*	*No data*	*No data*	*No data*	*No data*	*No data*	*No data*
**62**	Cirrhosis	2.8	11	*No data*	Male	Caucasian	63.0	NASH	1/29/2018	140	0.53	4.1	1.9	1.2	1	1	5	*No data*	*No data*	*No data*	*No data*	*No data*	*No data*	67	60	64	*No data*	*No data*	104	*No data*	*No data*	*No data*	*No data*	*No data*	*No data*	*No data*	*No data*	*No data*	*No data*
**63**	Cirrhosis	4.1	6	*No data*	Female	Caucasian	61.0	NASH	1/29/2018	147	0.59	4.1	0.8	1.0	1	1	5	*No data*	*No data*	*No data*	*No data*	*No data*	*No data*	20	24	54	*No data*	*No data*	218	*No data*	*No data*	*No data*	*No data*	*No data*	*No data*	*No data*	*No data*	*No data*	*No data*
**64**	Cirrhosis	1.7	10	*No data*	Female	Caucasian	76.0	NASH	2/5/2018	143	1.52	3.7	0.2	0.9	1	1	5	*No data*	*No data*	*No data*	*No data*	*No data*	*No data*	19	29	156	*No data*	*No data*	335	*No data*	*No data*	*No data*	*No data*	*No data*	*No data*	*No data*	*No data*	*No data*	*No data*
**65**	Cirrhosis	4.3	7	*No data*	Male	Caucasian	63.0	NASH	2/6/2018	138	0.71	3.3	0.9	1.1	1	1	6	*No data*	*No data*	*No data*	*No data*	*No data*	*No data*	20	32	102	*No data*	*No data*	244	*No data*	*No data*	*No data*	*No data*	*No data*	*No data*	*No data*	*No data*	*No data*	*No data*
**66**	Cirrhosis	4.0	9	*No data*	Female	Hispanic	62.0	NASH	2/8/2018	139	0.61	3.5	1.2	1.2	1	1	6	*No data*	*No data*	*No data*	*No data*	*No data*	*No data*	30	43	148	*No data*	*No data*	97	*No data*	*No data*	*No data*	*No data*	*No data*	*No data*	*No data*	*No data*	*No data*	*No data*
**68**	Cirrhosis	2.2	10	*No data*	Female	Caucasian	38.0	NASH	2/15/2018	138	1.07	4.2	0.3	1.3	1	2	6	*No data*	*No data*	*No data*	*No data*	*No data*	*No data*	24	22	113	*No data*	*No data*	194	*No data*	*No data*	*No data*	*No data*	*No data*	*No data*	*No data*	*No data*	*No data*	*No data*
**69**	Cirrhosis	2.0	7	*No data*	Female	Caucasian	52.0	NASH	2/16/2018	139	0.62	4.1	0.6	1.1	1	1	5	*No data*	*No data*	*No data*	*No data*	*No data*	*No data*	19	28	57	*No data*	*No data*	92	*No data*	*No data*	*No data*	*No data*	*No data*	*No data*	*No data*	*No data*	*No data*	*No data*
**70**	Cirrhosis	2.6	6	*No data*	Female	Caucasian	66.0	NASH	2/26/2018	143	0.81	4.2	0.5	1.0	1	1	5	*No data*	*No data*	*No data*	*No data*	*No data*	*No data*	46	48	146	*No data*	*No data*	115	*No data*	*No data*	*No data*	*No data*	*No data*	*No data*	*No data*	*No data*	*No data*	*No data*
**71**	Cirrhosis	4.0	6	*No data*	Female	Hispanic	49.0	NASH	3/6/2018	142	0.68	4.6	0.3	1.0	1	1	5	*No data*	*No data*	*No data*	*No data*	*No data*	*No data*	33	26	63	*No data*	*No data*	182	*No data*	*No data*	*No data*	*No data*	*No data*	*No data*	*No data*	*No data*	*No data*	*No data*
**73**	Cirrhosis	2.3	8	*No data*	Female	Caucasian	60.0	NASH	3/8/2018	142	0.61	3.9	0.4	1.2	1	1	5	*No data*	*No data*	*No data*	*No data*	*No data*	*No data*	34	31	92	*No data*	*No data*	92	*No data*	*No data*	*No data*	*No data*	*No data*	*No data*	*No data*	*No data*	*No data*	*No data*
**74**	Cirrhosis	6.0	9	*No data*	Female	Caucasian	66.0	NASH	3/19/2018	137	1.13	4.0	1.3	1.1	1	1	5	*No data*	*No data*	*No data*	*No data*	*No data*	*No data*	69	97	84	*No data*	*No data*	96	*No data*	*No data*	*No data*	*No data*	*No data*	*No data*	*No data*	*No data*	*No data*	*No data*
**75**	Cirrhosis	1.9	6	*No data*	Female	Asian	61.0	NASH	3/19/2018	140	0.78	4.5	0.4	1.0	1	1	5	*No data*	*No data*	*No data*	*No data*	*No data*	*No data*	82	84	69	*No data*	*No data*	215	*No data*	*No data*	*No data*	*No data*	*No data*	*No data*	*No data*	*No data*	*No data*	*No data*
**80**	HCC	2.5	14	1	Male	White	65.0	NASH	10/30/2017	*No data*	1.18	2.8	2.2	1.2	2	1	8	1	3.1	No	No	No	Yes	21	35	161	136	4.75	159	1.098	No	No	No	Quit	Quit (heavy hx)	Yes, on insulin	0	Yes	None
**81**	HCC	68.0	7	3	Male	White	65.1	NASH	10/13/2017	*No data*	0.66	3.8	1.0	1.1	1	1	5	2	10	Yes	No	No	Yes	121	85	184	142	9.57	168	-0.272	No	No	No	Quit	Quit (heavy hx)	Yes	0	No	One 1st degree relative
**82**	HCC	2221.0	30	3	Male	Black	78.2	NASH	10/5/2017	*No data*	7.47	2.5	2.3	1.9	3	1	11	98	NA	Yes	No	No	*No data*	38	169	147	138	9.32	118	1.383	Yes	No	No	Quit	Quit (heavy hx)	Yes, on insulin	3	No	None
**83**	HCC	4.6	7	3	Female	White	58.9	NASH	10/6/2017	*No data*	0.90	3.8	0.4	1.1	1	1	5	>5	15	Yes	No	No	Yes	50	71	308	144	11.90	493	-0.877	Yes	No	No	None	None	Yes, on insulin	0	No	None
**84**	HCC	52173.0	11	4	Female	White	59.6	NASH	11/16/2017	*No data*	0.60	2.8	1.9	1.1	1	1	6	98	NA	Yes	No	Yes	Yes	78	195	698	133	7.80	282	1.002	Yes	No	No	None	None	*No data*	2	No	None
**85**	HCC	6.0	11	3	Female	Hispanic	43.4	NASH	12/8/2018	*No data*	0.49	1.9	1.9	1.2	2	1	6	2	9.6	No	No	No	Yes	66	103	130	132	9.20	167	1.767	No	No	No	*No data*	Active (occasional/social)	*No data*	0	No	None
**86**	HCC	60500.0	14	4	Male	Hispanic	47.2	NASH	1/5/2018	*No data*	0.72	3.6	0.7	1.6	1	1	5	2	17.2	Yes	Yes	Yes	Yes	29	46	146	135	7.69	200	-0.337	Yes	Yes	Lung/pancreas	Quit	Quit (heavy hx)	*No data*	0	No	None
**87**	HCC	3.2	10	1	Female	Other	65.8	NASH	12/12/2017	*No data*	1.00	3.7	2.1	1.1	1	2	7	1	2.5	No	No	No	Yes	49	48	225	150	3.95	166	0.303	No	No	No	None	None	Yes, on insulin	0	Yes	None
**88**	HCC	7.0	21	1	Female	White	73.8	NASH	1/19/2018	*No data*	1.53	3.6	0.3	2.5	1	1	7	1	2.7	No	No	No	Yes	23	31	83	139	10.48	192	-0.897	No	No	No	None	None	Yes, on insulin	0	*No data*	None
**89**	HCC	310.4	9	1	Female	White	75.3	NASH	3/6/2018	*No data*	0.59	3.3	0.6	1.3	1	1	6	1	3	No	No	No	Yes	30	55	87	137	5.52	70	-0.184	No	No	No	None	None	Yes, on insulin	0	No	None
**91**	HCC	5.3	8	3	Male	White	60.0	NASH	3/25/2018	*No data*	0.83	3.3	1.4	1.0	2	1	7	98	NA	No	No	No	Yes	60	55	120	138	10.25	465	0.375	No	No	No	Quit	Quit (heavy hx)	*No data*	*No data*	No	None
**92**	HCC	4.0	8	3	Female	Hispanic	64.0	NASH	4/13/2018	*No data*	0.83	3.2	0.6	1.2	1	1	6	1	8.6	Yes	No	No	Yes	24	48	274	137	7.36	152	-0.099	No	No	No	Quit	None	*No data*	*No data*	No	None
**94**	HCC	4.6	9	1	Male	White	59.2	NASH	6/5/2018	*No data*	0.78	3.9	0.8	1.3	2	1	6	1	4.9	No	No	No	Yes	48	59	168	134	4.72	67	-0.504	No	No	No	Quit	Quit (heavy hx)	Yes, on insulin	0	No	None
**95**	HCC	12.0	21	1	Female	Hispanic	68.4	NASH	6/8/2018	*No data*	2.53	2.6	2.3	1.3	2	2	10	2	3.6	No	No	No	Yes	20	35	79	138	3.86	91	1.298	No	No	No	None	None	Yes, on insulin	*No data*	No	None
**96**	HCC	3.0	6	1	Male	Hispanic	63.4	NASH	7/13/2018	*No data*	0.79	4.5	0.4	1.0	1	1	5	1	2.5	No	No	No	No	42	27	59	142	7.54	213	-1.472	No	No	No	None	None	Yes, on metformin	*No data*	No	None

Supplementary Table S4. Descriptive statistics of haptoglobin *N*-glycopeptides with important changes between cirrhosis and HCC samples. $MVSHHN_{184}LTTGATLINE$ = Asn184, $NLFLN_{207}HSE$ = Asn207, $VVLHPN_{241}YSQVDIGLIK$ = Asn241, and AFP = alpha-fetoprotein. Glycan nomenclature: HexNAc, Hex, Fuc, NeuAc (*N*-acetylhexosamine, Hexose, Fucose, *N*-acetylneuraminic acid).

Variable	Diagnosis	Sample number	Relative response (%)		*p* value
			Mean	Standard deviation	
AFP	Cirrhosis	15	3.11	1.59	**0.001**
	HCC	15	7688.31	19821.80	
Asn184 + 4-5-1-2	Cirrhosis	15	0.94	0.42	**0.131**
	HCC	15	1.64	1.21	
Asn184 + 5-6-1-3	Cirrhosis	15	0.38	0.39	**0.077**
	HCC	15	0.83	0.63	
Asn184 + 5-6-0-2 Isomer 2	Cirrhosis	15	0.67	0.34	**0.128**
	HCC	15	0.41	0.33	
Asn207 + 4-4-0-1 Isomer 3	Cirrhosis	15	0.01	0.01	**0.132**
	HCC	15	0.02	0.02	
Asn207 + 4-5-0-0 Isomer 2	Cirrhosis	15	0.16	0.07	**0.104**
	HCC	15	0.11	0.05	
Asn207 + 5-6-1-2 Isomer 2	Cirrhosis	15	2.43	1.55	**0.012**
	HCC	15	4.23	1.59	
Asn207 + 5-6-1-3 Isomer 1	Cirrhosis	15	3.51	1.94	**0.001**
	HCC	15	6.85	2.48	
Asn207 + 5-6-1-3 Isomer 2	Cirrhosis	15	0.50	0.29	**0.005**
	HCC	15	0.96	0.42	
Asn207 + 5-6-0-1 Isomer 2	Cirrhosis	15	2.75	1.64	**0.010**
	HCC	15	1.31	0.59	
Asn207 + 5-6-0-2 Isomer 1	Cirrhosis	15	3.67	1.09	**0.038**
	HCC	15	2.70	0.89	
Asn207 + 5-6-0-2 Isomer 2	Cirrhosis	15	7.11	1.78	**0.033**
	HCC	15	5.16	2.12	
Asn207 + 6-7-1-1 Isomer 3	Cirrhosis	15	0.08	0.11	**0.085**
	HCC	15	0.02	0.02	
Asn207 + 6-7-1-2 Isomer 1	Cirrhosis	15	0.19	0.15	**0.096**
	HCC	15	0.47	0.46	
Asn207 + 6-7-1-2 Isomer 3	Cirrhosis	15	0.05	0.02	**0.014**
	HCC	15	0.08	0.02	
Asn207 + 6-7-1-3 Isomer 5	Cirrhosis	15	0.07	0.07	**0.094**
	HCC	15	0.13	0.08	
Asn241 + 4-5-0-0	Cirrhosis	15	0.03	0.03	**0.050**
	HCC	15	0.01	0.01	
Asn241 + 4-5-0-1	Cirrhosis	15	5.73	0.95	**0.065**
	HCC	15	4.95	0.78	
AFP (**early HCC**)*	Cirrhosis	7	3.10	0.9	**0.354**
	HCC	7	48.96	115.33	
Asn207 + 5-6-0-1 Isomer 2 (**early HCC**)*	Cirrhosis	7	3.88	1.61	**0.006**
	HCC	7	1.43	0.42	

*** Cirrhosis samples; NASH60, 61, 62, 65, 66, 70, and 75. Early HCC samples; NASH80, 87, 88, 89, 94, 95, and 96.**
***p* – value obtained from T – Test and corrected using Bonferroni Correction.**

Supplementary Table S5. Determination of haptoglobin *N*-glycopeptides with significative changes in abundance between cirrhosis and HCC using same gender ratio female : male in both sample cohorts (p value <0.05). MVSHHN$_{184}$LTTGATLINE = Asn184, NLFLN$_{207}$HSE = Asn207, VVLHPN$_{241}$YSQVDIGLIK = Asn241, and AFP = alpha-fetoprotein. Glycan nomenclature: HexNAc, Hex, Fuc, NeuAc (*N*-acetylhexosamine, Hexose, Fucose, *N*-acetylneuraminic acid).

***N*-Glycopeptides**	**Samples:** **15 Cirrhosis** **15 HCC**	**Samples:** **7 Cirrhosis; M/F* (43/57 %)** **15 HCC ; M/F (43/57 %)** **For each test different female cirrhosis samples were used*	
**COMPARISON**	**1**	**3**	**4**
**MVSHHN$_{184}$LTTGATLINE**	***p* value** [A, C]	***p* value**[B, C]	***p* value**[B, C]
Asn184 + 4-5-1-2	0.131	0.560	0.656
Asn184 + 5-6-1-3	0.077	0.553	0.379
Asn184 + 5602 Isomer 2	0.128	0.048	0.368
**NLFLN$_{207}$HSE**			
Asn207 + 4-4-0-1 Isomer 3	0.132	1.000	0.513
Asn207 + 4-5-0-0 Isomer 2	0.104	0.029	0.242
Asn207 + 5-6-1-2 Isomer 2	0.012	0.059	0.123
Asn207 + 5-6-1-3 Isomer 1	0.001	0.027	0.048
Asn207 + 5-6-1-3 Isomer 2	0.005	0.048	0.050
Asn207 + 5-6-0-1 Isomer 2	0.010	0.001	0.006
Asn207 + 5-6-0-2 Isomer 1	0.038	0.016	0.018
Asn207 + 5-6-0-2 Isomer 2	0.033	0.113	0.051
Asn207 + 6-7-1-1 Isomer 3	0.085	0.063	0.010
Asn207 + 6-7-1-2 Isomer 1	0.096	0.516	0.404
Asn207 + 6-7-1-2 Isomer 3	0.014	0.504	1.014
Asn207 + 6-7-1-3 Isomer 5	0.094	0.825	0.934
**VVLHPN$_{241}$YSQVDIGLIK**			
Asn241 + 4-5-0-0	0.050	0.142	0.121
Asn241 + 4-5-0-1	0.065	0.620	0.698

Comparison 1. Cirrhosis samples; NASH58, 60, 61, 62, 63, 64, 65, 66, 68, 69, 70, 71, 73, 74 and 69.
HCC samples; NASH80, 81, 82, 83, 84, 85, 86, 87, 88, 89, 91, 92, 94, 95 and 96.
Comparison 2. Cirrhosis samples; NASH60, 61, 62, 65, 66, 68, and 71.
Comparison 3. Cirrhosis samples; NASH60, 62, 65, 71, 73, 74, and 75.
[A] ***p* – value obtained from T – Test.**
[B] ***p* – value obtained from Wilcoxon test.**
[C] ***p* – values were corrected according to Bonferroni Correction (n = 3, α = 0.05).**

Initially, we observe seventeen haptoglobin *N*-glycopeptides with significant changes in abundance between cirrhosis and HCC samples (Supplementary Table S4). However, the cirrhosis cohort have a large number of female samples. Therefore, to avoid gender bias, the performance of the significant *N*-glycopeptides was evaluated by using a constant female : male (57:43 %) ratio for both cohorts. To complete the evaluation three cirrhosis sample groups were tested, different female cirrhosis samples were selected for each evaluation (Supplementary Table S5). The large number of parameter used in this evaluation can increase the probability that the observed results are accidental. Therefore, the "Bonferroni Correction" was applied to the observed p values. The corrected p values can be observed in supplementary table S5. Despite the applied correction the

N-glycopeptides Asn207 + 5-6-1-3 Isomer 1, Asn207 + 5-6-1-3 Isomer 2, Asn207 + 5-6-0-1 Isomer 2, and the Asn207 + 5-6-0-2 Isomer 1 have p values lower than 0.05 in all tested sample groups. By using this evaluation we intended to increase the accuracy of the presented results in the main manuscript.

Supplementary Table S6. Statistical comparison between gender groups. NLFLN$_{207}$HSE = Asn207, VVLHPN$_{241}$YSQVDIGLIK = Asn241, and AFP = alpha-fetoprotein. Glycan nomenclature: HexNAc, Hex, Fuc, NeuAc (*N*-acetylhexosamine, Hexose, Fucose, *N*-acetylneuraminic acid).

To prove that the *N*-glycopeptides selected to compare the cirrhosis and HCC were not biased by the gender groups the following statistical analysis was completed (a, b, and c).

a) Comparison of the statistically significant N-glycopeptides between gender groups.

Variable	Gender[1,2]	N	Min	Median	Max	Mean	SD	*p* value[3]
Asn207 + 5-6-1-3 Isomer 1	Female	20	1.34	3.97	11.00	4.46	2.62	0.035
	Male	10	1.32	7.01	10.41	6.62	2.61	
Asn241 + 4-5-0-0	Female	20	0.00	0.02	0.12	0.02	0.03	0.091
	Male	10	0.00	0.01	0.02	0.01	0.01	
Asn207 + 5-6-1-2 Isomer 2	Female	20	0.84	2.56	5.59	2.92	1.68	0.169
	Male	10	0.96	4.14	7.37	4.14	1.84	
Asn207 + 5-6-1-3 Isomer 2	Female	20	0.17	0.53	1.82	0.70	0.48	0.287
	Male	10	0.15	0.78	1.13	0.78	0.30	
Asn207 + 6-7-1-1 Isomer 3	Female	20	0.01	0.02	0.42	0.06	0.10	0.328
	Male	10	0.00	0.02	0.12	0.03	0.04	
Asn207 + 5-6-0-1 Isomer 2	Female	20	0.33	1.80	5.69	2.21	1.54	0.422
	Male	10	0.42	1.52	3.52	1.67	1.11	
Asn207 + 5-6-0-2 Isomer 1	Female	20	1.50	3.32	6.30	3.28	1.08	0.746
	Male	10	1.24	3.24	5.00	3.00	1.15	

[1] Female samples; NASH58, 61, 63, 64, 66, 68, 69, 70, 71, 73, 74, 75, 83, 84, 85, 87, 88, 89, 92, and 95.
[2] Male samples; NASH60, 62, 65, 80, 81, 82, 86, 91, 94, and 96.
[3] *p* – value obtained from Wilcoxon test.

Based on the Wilcoxon test, only Asn207 + 5-6-1-3 Isomer 1 had statistically significant difference between gender groups, and Asn241 + 4-5-0-0 showed marginally significant difference between gender groups.

We have added gender to the single and group glycopeptide panels, the estimated AUC and its 95% CI were summarized in b). We also completed the corresponding logistic regression model for the evaluated glycopeptides, see evaluation in c).

b) Multiple-variable model adding gender. Single and group glycopeptide models, differentiation of cirrhosis and HCC.

Glycopeptide	AUC	CI[1] (low)	CI[1] (high)
[Asn207 + 5-6-1-3 Isomer 1]+gender	0.86	0.69	0.97
[Asn241 + 4-5-0-0]+gender	0.79	0.62	0.93
AFP + [Asn207 + 5-6-1-3 Isomer 1] +gender	0.95	0.86	1.00

[1] Confidence interval (95% CI).

c) The corresponding logistic regression model.

Model	Variable	Coefficient	*p* value
1	Asn207 + 5-6-1-3 Isomer 1	0.66	0.012
	Gender	0.16	0.887
2	Asn241 + 4-5-0-0	-98.72	0.030
	Gender	0.48	0.611
3	AFP	0.77	0.044
	Asn207 + 5-6-1-3 Isomer 1	0.55	0.039
	Gender	1.36	0.337

The similarity of the AUC results when gender is added to the evaluation and the initial values (Table 2 in the manuscript). It was obvious that adding gender to the panels did not change the performance evidence by similar estimated AUC. The effect of gender in all the three logistic models was also not statistically significant. Therefore, no significant bias was introduced by gender.

Supplementary Table S7. Determination of haptoglobin *N*-glycopeptides with significative changes in abundance between cirrhosis and **early HCC** using same gender ratio female : male in both sample cohorts. $MVSHHN_{184}LTTGATLINE$ = Asn184, $NLFLN_{207}HSE$ = Asn207, $VVLHPN_{241}YSQVDIGLIK$ = Asn241, and AFP = alpha-fetoprotein. Glycan nomenclature: HexNAc, Hex, Fuc, NeuAc (*N*-acetylhexosamine, Hexose, Fucose, *N*-acetylneuraminic acid).

***N*-Glycopeptides**	**Samples:**<br>**7 Cirrhosis; M/F* (42.9%/57.1%)**<br>**7 EARLY HCC; M/F (42.9%/57.1%)**<br>**For each test different female cirrhosis samples were used*		
**COMPARISON**	**1**	**2**	**3**
**$MVSHHN_{184}LTTGATLINE$**	***p* value$^{A, B}$**	***p* value$^{A, B}$**	***p* value$^{A, B}$**
Asn184 + 4-5-1-2	1.000	1.000	0.746
Asn184 + 5-6-1-3	0.667	0.965	0.415
Asn184 + 5602 Isomer 2	0.191	0.119	0.440
**$NLFLN_{207}HSE$**			
Asn207 + 4-4-0-1 Isomer 3	1.000	1.000	0.679
Asn207 + 4-5-0-0 Isomer 2	0.173	0.554	0.350
Asn207 + 5-6-1-2 Isomer 2	0.241	0.376	0.375
Asn207 + 5-6-1-3 Isomer 1	0.159	0.234	0.234
Asn207 + 5-6-1-3 Isomer 2	0.192	0.630	0.334
Asn207 + 5-6-0-1 Isomer 2	0.006	0.034	0.040
Asn207 + 5-6-0-2 Isomer 1	0.032	0.050	0.175
Asn207 + 5-6-0-2 Isomer 2	0.322	0.670	0.600
Asn207 + 6-7-1-1 Isomer 3	0.318	1.000	0.147
Asn207 + 6-7-1-2 Isomer 1	0.702	1.000	0.394
Asn207 + 6-7-1-2 Isomer 3	0.750	0.199	0.560
Asn207 + 6-7-1-3 Isomer 5	1.000	1.000	1.000
**$VVLHPN_{241}YSQVDIGLIK$**			
Asn241 + 4-5-0-0	0.527	0.338	0.751
Asn241 + 4-5-0-1	1.000	0.567	1.000

Comparison 1. Cirrhosis samples; NASH60, 61, 62, 65, 66, 70, and 75.
Comparison 2. Cirrhosis samples; NASH60, 61, 62, 64, 65, 68, and 70.
Comparison 3. Cirrhosis samples; NASH60, 62, 65, 66, 68, 73, and 74.
^{A} *p* – value obtained from T – Test.
^{B} *p* – values were corrected according to Bonferroni Correction ($n = 3$, $\alpha = 0.05$).

The efficacy of the initial seventeen haptoglobin *N*-glycopeptides (Supplementary Table S4) was tested for the HCC samples in TNM 1 stage (early HCC). To avoid gender bias, the performance of the *N*-glycopeptides was evaluated by using a constant female : male (57:43 %) ratio for both cohorts. To complete the evaluation three cirrhosis sample groups were tested, different female cirrhosis samples were selected for each evaluation (Supplementary Table S7). The large number of parameter used in this evaluation can increase the probability that the observed results are accidental. Therefore, the "Bonferroni Correction" was applied to the observed *p* values. The corrected *p* values can be observed in supplementary table S7. Despite the applied correction the *N*-glycopeptide Asn207 + 5-6-0-1 Isomer 2 has *p* values lower than 0.05 in

all tested sample groups. By using this evaluation we intended to increase the accuracy of the presented results in the main manuscript.
